# A Bayesian Shrinkage Approach for AMMI Models

**DOI:** 10.1371/journal.pone.0131414

**Published:** 2015-07-09

**Authors:** Carlos Pereira da Silva, Luciano Antonio de Oliveira, Joel Jorge Nuvunga, Andrezza Kéllen Alves Pamplona, Marcio Balestre

**Affiliations:** 1 Department of Exact Science (DEX), Federal University of Lavras, Lavras, Minas Gerais, Brazil; 2 Faculty of Exact Sciences and Technology (FACET), Federal University of Grande Dourados, Grande Dourados, Mato Grosso do Sul, Brazil; National Taiwan University, TAIWAN

## Abstract

Linear-bilinear models, especially the additive main effects and multiplicative interaction (AMMI) model, are widely applicable to genotype-by-environment interaction (GEI) studies in plant breeding programs. These models allow a parsimonious modeling of GE interactions, retaining a small number of principal components in the analysis. However, one aspect of the AMMI model that is still debated is the selection criteria for determining the number of multiplicative terms required to describe the GE interaction pattern. Shrinkage estimators have been proposed as selection criteria for the GE interaction components. In this study, a Bayesian approach was combined with the AMMI model with shrinkage estimators for the principal components. A total of 55 maize genotypes were evaluated in nine different environments using a complete blocks design with three replicates. The results show that the traditional Bayesian AMMI model produces low shrinkage of singular values but avoids the usual pitfalls in determining the credible intervals in the biplot. On the other hand, Bayesian shrinkage AMMI models have difficulty with the credible interval for model parameters, but produce stronger shrinkage of the principal components, converging to GE matrices that have more shrinkage than those obtained using mixed models. This characteristic allowed more parsimonious models to be chosen, and resulted in models being selected that were similar to those obtained by the Cornelius F-test (*α* = 0.05) in traditional AMMI models and cross validation based on leave-one-out. This characteristic allowed more parsimonious models to be chosen and more GEI pattern retained on the first two components. The resulting model chosen by posterior distribution of singular value was also similar to those produced by the cross-validation approach in traditional AMMI models. Our method enables the estimation of credible interval for AMMI biplot plus the choice of AMMI model based on direct posterior distribution retaining more GEI pattern in the first components and discarding noise without Gaussian assumption as requested in F-based tests or deal with parametric problems as observed in traditional AMMI shrinkage method.

## Introduction

Plant breeding programs select and recommend the best genotypes or cultivars based on yield and on their adaptability to and stability in various test environments. However, the breeder’s job is hindered by genotype-by-environment interactions *(GEI)*, a phenomenon that can be detected through the responses of the genotypes when evaluated in different environments.

Therefore, studies on *GEI* are of utmost importance, and can be performed using several different methods, including linear-bilinear models [1], which offer an improved description of the effects of interaction among factors. The additive main effects and multiplicative interaction (AMMI) model is currently one of the most popular multiplicative models. The AMMI model was originally proposed by Gollob [2] and Mandel [3,4] in the context of fixed effects, but the actual statistical method itself goes back to work by Pike and Silverberg [5] and Williams [6]. It has been widely used to study *GE* interactions in plant breeding programs and in agronomic experiments in general because it incorporates univariate and multivariate procedures to separate the pattern responsible for the interaction from the noise, which is an unexplained factor. The noise, which is of no agronomic interest, is removed to increase the predictive power of the model [7].

One aspect that is still debated in the literature is the method used to select the number of multiplicative terms necessary to describe the interaction pattern. Bilinear terms of the model have been selected using several different criteria. These include approximate F-tests, notably the F-test from Gollob [2] and the *F*_*R*_-test from Cornelius et al. [8], and non-parametric methods based on computationally intensive resampling, such as cross validation [7,9,10,11] and parametric bootstrapping, described by Forkman and Piepho [12].

An alternative for selecting the AMMI model multiplicative components has been described by Cornelius and Crossa [10,13]. These authors have proposed that an estimator that promotes shrinkage of the least squares estimates of the scale parameters in multiplicative models, specifically the singular values, be applied. In addition to obtaining more realistic estimates for the parameters, their method eliminates the need for any statistical tests or cross-validation methods to select components that describe the interaction pattern among factors. Additionally, the shrinkage estimators from Cornelius and Crossa [10] produced fitted models that were better than those fitted using approximate F-tests, and they were generally as good as or better than the best linear unbiased predictors (BLUPs) obtained from random models. Their approach was based on the idea that the phenotypic means obtained by the ordinary least squares (OLS) method are not the best at minimizing the mean squared error (MSE) and cannot be used in all situations [14].

Despite the above-mentioned advantages, the method proposed by Cornelius and Crossa [10] does not have a very clear derivation and may provide inconsistent results in the spectral decomposition, for example, negative singular values and magnitude inversion between high-dimensional and low-dimensional singular values. Additionally, the method proposed by these authors can only be used to predict the genotypic value of a genotype present in a specific environment, i.e., it is applied in the context of models with fixed effect parameters and, therefore, requires balanced data and homogeneous variance, which cannot always be guaranteed for data from multi-environment trials (MET). Furthermore, many conclusions about adaptability and stability in multiplicative models are obtained from biplots [15] of the genotype and environment scores that generally do not involve any measure of uncertainty or use inferential procedures prone to criticism, either from the assumptions required for parametric methods or by problematic procedures used in bootstrap resampling [16,17]. Missing data and heterogeneous variance are not large problems for factor analyses equivalent to AMMI models and sites regression (SREG) models, proposed by Burgueño et al. [18], Piepho [19] and Smith et al.[20], that also allow the GEI to be random. However, as Crossa et al. [21] have shown, it is not clear how the confidence regions used in fixed effect models can be effectively incorporated in BLUPs in multiplicative mixed models for interaction parameters.

An alternative for complete analysis of MET data is to use a Bayesian approach for the AMMI model fit, as proposed by Crossa et al. [21] and Perez—Elisald et al. [22]. Viele and Srinivazan [23] were the first to use an inference method with the AMMI model in a Bayesian context, using Markov chain Monte Carlo (MCMC) methods. These authors showed as sampling bilinear parameters of model when the support for the joint posterior distribution was nontrivial. Liu [24] developed a set of conditional posteriors where sampling for the parameters can be performed directly using a Gibbs sampler, which resulted in a more stable algorithm. The flexibility to include credible regions in the biplot and information in the inference process was demonstrated by Crossa et al. [21], Perez—Elisald et al. [22], Forkman and Piepho [12] and Oliveira et al. [25]. According to these authors, in addition to all of these advantages, Bayesian maximum a posteriori (MAP) estimators for singular values produce shrinkage similar to those observed by Crossa et al. [21], even with uninformative priors for the truncated normal with high variance. The result is a MAP estimator analytically similar to an OLS estimator, but empirically similar to the shrinkage estimator. Therefore, an analysis that effectively explores the analytical properties of shrinkage estimators using Bayesian inference should be developed.

This study proposed singular value shrinkage estimators for AMMI models using the Bayesian shrinkage approach. A shrinkage estimator, similar to the one proposed by Cornelius and Crossa [10], was analytically justified and the results were compared with conventional Bayesian AMMI analysis.

## Materials and Methods

To illustrate the proposed method, a dataset using 55 maize hybrids evaluated in nine different environments for grain yield, in *kg*.*ha* ^-1^, was employed. The experimental design was a complete block design with three replicates and evaluated to rows that were 3m in length during the agricultural year 2005 to 2006 (Table 1).

**Table 1:** Characteristics of the experimental environments.

Environment	Municipality	Latitude	Longitude	CV^1^ (%)	Mean yield (t ha^-1^)
**E1**	Lavras, MG	21°13´S	44°58´W	14.1	10.803
**E2**	Guarda-Mor, MG	17°34´S	47°08´W	13.5	6.212
**E3**	Barreiras, BA	12°08´S	45°00´W	12.0	4.549
**E4**	Jussara, GO	23°35´S	52°28´W	12.5	5.152
**E5**	Lavras, MG	21°12´S	44°58´W	20.8	6.246
**E6**	São Gotardo, MG	19°18´S	46°03´W	11.3	8.085
**E7**	Ijaci, MG	21°09´S	44°56´W	10.1	13.192
**E8**	Ijaci, MG	21°10´S	44°56´W	10.7	8.896
**E9**	Lavras, MG	21°10´S	45°03´W	14.6	8.737

Source: Machado et al., 2007.

^1^coefficient of variation.

### AMMI model and shrinkage effect for singular values in the context of fixed effects

The traditional AMMI model for fixed effects, with *y*_*ij*_, *i* genotypes (*i* = 1, 2,…, *g*) and *j* environments (*j* = 1, 2,…, *a*), is described as follows:

$$y_{ij}=\mu +\tau_{i}+\beta_{j}+\sum_{k=1}^{t}\lambda_{k}\alpha_{ik}\gamma_{jk}+\epsilon_{ij}$$ (1)

where *μ* is the overall mean of the experiment, *τ*_*i*_ is the effect of the *i*th genotype, *β*_*j*_ is the effect of the *j*th environment, *λ*_*k*_ is the *k*th singular value of the multiplicative component, *α*_*ik*_ is the *i*th element of the *k*th genotypic singular vector, *γ*_*jk*_ is the *j*th element of the *k*th environment singular vector, *e*_*ij*_ is the error associated with the *i*th genotype of the *j*th environment, and *t* **≤**min(*g*-1, *a*-1).

This model also has the restrictions of identifiability $\left(\sum_{i}\tau_{i}=\sum_{j}\beta_{j}=\sum_{i}{\left(\tau \beta \right)}_{ij}=\sum_{j}{\left(\tau \beta \right)}_{ij}=0\right)$, orthonormality of the singular vectors $(\sum_{i}\alpha_{ik}^{2}=\sum_{i}\gamma_{jk}^{2}=1,\;\sum_{i}\alpha_{ik}\alpha_{ik^{\prime }}=\sum_{j}\gamma_{jk}\gamma_{jk^{\prime }}=0,\;k^{\prime }\neq k)$ and that *λ*_*k*_ should satisfy the condition *λ*_*k*_ ≥ *λ*_*k*+1_, for any *k* > 0.

#### Shrinkage effect for fixed effect models

Cornelius and Crossa [10] proposed that a shrinkage estimator be used for the bilinear terms to study *GEI* in multiplicative models, notably in AMMI models. The shrinkage estimator assumes that each empirical bilinear least squares component is the “true” value of the effect plus a disturbance (*η*_*ijk*_), i.e., ${\hat{\lambda }}_{k}{\hat{\alpha }}_{ik}{\hat{\gamma }}_{jk}=\lambda_{k}\alpha_{ik}\gamma_{jk}+\eta_{ijk}$, such that *λ*_*k*_ *α*_*ik*_ *γ*_*jk*_ and *η*_*ijk*_ are not correlated, and *λ*_*k*'_ *α*_*ik*'_ *γ*_*jk*'_ and *η*_*ijk*_ are not correlated, for *k* ≠ *k*'. The shrinkage estimator $\sum_{k}S_{k}{\hat{\lambda }}_{k}{\hat{\alpha }}_{ik}{\hat{\gamma }}_{jk}$ estimates the contribution of Σ_*k*_ *λ*_*k*_ *α*_*ik*_ *γ*_*jk*_ to the mean of the observed plot *μ*_*ij*_, and the average quadratic estimation error per plot for the AMMI model is minimized if $S_{k}=E(\lambda_{k}^{2})/E({\hat{\lambda }}_{k}^{2})\;.$ According to these authors the $E(\lambda_{k}^{2})$ means the expectation of estimated lambda on the true value. Defining *u*_*k*_ such that $E\left(\sum_{i}\sum_{j}\eta_{ijk}^{2}\right)=u_{k}\sigma^{2}/n\;,$ where *σ* ^2^ is the residual variance and n the sample size yields $E\left(\sum_{i}\sum_{j}\eta_{ijk}^{2}\right)=E({\hat{\lambda }}_{k}^{2})-E(\lambda_{k}^{2})$ and, consequently,$S_{k}=\;1\text{ -}\frac{u_{k}\sigma^{2}/n}{E({\hat{\lambda }}_{k}^{2})}$ , where $\frac{u_{k}\sigma^{2}/n}{E({\hat{\lambda }}_{k}^{2})}$ is similar to an approximate *F* ^*-1*^-test. Furthermore, $S_{k}=max(1-F_{k}^{-1},0)$, the shrinkage factor in this method, can be negative.

The authors showed that, occasionally,$S_{k}{\hat{\lambda }}_{k-1}>S_{k-1}{\hat{\lambda }}_{k-1}$ , which violates the model restriction *λ*_*k*_ ≤ *λ*_*k*-1_. To overcome this problem, it is assumed that ${\hat{\lambda }}_{k}={\hat{\lambda }}_{k-1}$ and a joint estimate was calculated. The rule was applied sequentially; it also yielded joint estimates for sequences of more than two singular values.

Although it violates several critical restrictions of the analysis, models fitted by this method are more parsimonious than those based on the least squares method, and, in general, the shrinkage estimates were as good as or better than the predictions made by random models. This method is based on minimizing the MSE and follows the principle proposed by Stein [26]. Despite all of the advantages of this method, it is naturally subject to the limitations of models conceived in the context of fixed effects.

### Bayesian shrinkage AMMI model

The AMMI model described at Eq 1 is related to a two-way table. In order to expand the AMMI effect to plot level at any environment we can to describe the model 1 in matrix form, with confounded block and environment effects represented as follows:

$$y=\;X_{1}\beta +\;Z\delta +\sum_{k=1}^{t}\lambda_{k}diag(Z\alpha_{k})X_{2}\gamma_{k}+\epsilon$$ (2)

where **y** is the vector of observations with dimension *n*×1, **X**_1_ is the design matrix with dimension *n*×*a* associated with the block plus environments effects vector **β**, **Z** is a design matrix with dimension *n*×*g* associated with the genotypic parameter vector δ with dimension *g*×1, *λ*_*k*_ is the kth singular value under the conditions mentioned previously, **α**_*k*_ and **γ**_*k*_ are the kth eigenvectors for the genotype and the environment, respectively, and ϵ is the vector of the residuals with multivariate normal probability distribution with mean of zero and variance and covariance matrix $I\sigma_{e}^{2}$. The matrix representation of inner product *diag*(**Zα**_*k*_)**X**_2_ **γ**_*k*_ is used in order to account for GEI in data level where **X**_2_ account only for environments design. More detail can be obtained in the R program available in S1 Text—line 63).

#### Prior distribution for the model parameters

A prior distribution represents the previous knowledge about a parameter of interest. This prior knowledge can be informative or uninformative, depending on the currently available knowledge about the phenomenon or on the amount of information needed for the model, which are set a priori using hyperparameter values.

Therefore, determining the prior distribution for the effects of the singular values defines the Bayesian shrinkage model. In this case, the prior distributions for AMMI model (2) assigned to the parameters were as follows:

p(β)=1/σ=k (constant)

$$\delta |\mu_{\delta },\sigma_{\delta }^{2}\sim N(0,I\sigma_{\delta }^{2})$$

$$\lambda_{k}|\mu_{\lambda_{k}},\sigma_{\lambda_{k}}^{2}\sim N^{+}(0,\sigma_{\lambda_{k}}^{2})$$

Where **β** is the fixed effects $\mu_{\delta }\;and\;\sigma_{\delta }^{2}$ are mean and variance hypothesis parameters related to effect of genotypes, $\mu_{\lambda_{k}},\;and\;\sigma_{\lambda_{k}}^{2}$ are mean and variance hypothesis parameters related to kth singular value and *N* ^+^ means a normal truncated. The non informative priors for singular vector related to genotypes and environments are respectively given by:

**α**_*k*_~ uniform spherical distribution in the correct subspace *(SUNI*_*d*_ *)*
**γ**_*k*_~ uniform spherical distribution in the correct subspace *(SUNI*_*d*_ *)*.
$\sigma_{\delta }^{2}\sim Inv-scaled-\chi^{2}(\upsilon_{\delta },S_{\delta }^{2})\propto \frac{1}{\sigma_{\delta }^{2}}$ for *υ*_*δ*_ = 0 and $S_{\delta }^{2}=0$
$\sigma_{e}^{2}\sim Inv-scaled-\chi^{2}(\upsilon_{e},S_{e}^{2})\propto \frac{1}{\sigma_{e}^{2}}$ for *υ*_*e*_ = 0 and $S_{e}^{2}=0$

where *Inv*-*scaled*-*χ* ^2^ is the inverted-scaled chi-square distribution with *υ* degree of freedom and *S* ^2^ scale parameter. The priors for **β** $\sigma_{\delta }^{2}$ and $\sigma_{e}^{2}$ are equivalent to Jeffrey priors that can be obtained by the expected Fisher Information of the likelihood distribution (given below). For the singular vectors **α**_*k*_ and **γ**_*k*_, a uniform spherical distribution prior, which are uninformative and special cases of the von Mises-Fisher distribution [27], were used. These vectors are distributed in a restricted space in ℜ^*p*^ orthogonal to *t*-1 vectors in the space of dimension *p* (*p = g* or *p = a*).

Given that $\sigma_{\lambda_{k}}^{2}$ is an unknown parameter in $p(\lambda_{k}|\mu_{\lambda_{k}},\sigma_{\lambda_{k}}^{2})$, it must be estimated, the modeling of this effect is included in the joint prior. The prior distribution for this parameter is the same as that described for $\sigma_{e}^{2}$ and $\sigma_{\delta }^{2}$, i.e., an uninformative prior was used with the property $p(\sigma_{\lambda_{k}}^{2})={\left(\sigma_{\lambda_{k}}^{2}\right)}^{-1}$.

Analytically, this prior resulted in an improper marginal posterior distribution, also verified by Ter Braak et al. [28] in shrinkage models with specific variances. To correct this problem, the extended prior presented by Ter Braak et al. [28] was adopted, correcting the degrees of freedom to obtain a proper posterior distribution. Therefore, the prior for the specific variance of *λ*_*k*_ is as follows: Assuming $S_{\lambda_{k}}^{2}=0$ and $\upsilon_{\lambda_{k}}=\;n_{\lambda_{k}}-\;1\;=\;-2\Delta$ we have:

$$\begin{array}{l} p\left(\sigma_{\lambda_{k}}^{2}\right)\propto {\left(\sigma_{\lambda_{k}}^{2}\right)}^{-\frac{\left(n_{\lambda_{k}}-1\right)}{2}-1}exp\left\{\frac{S_{\lambda_{k}}^{2}}{2\sigma_{\lambda_{k}}^{2}}\right\}\\ p\left(\sigma_{\lambda_{k}}^{2}\right)\propto {\left(\sigma_{\lambda_{k}}^{2}\right)}^{-\frac{\left(-2\Delta \right)}{2}-1}exp\left\{\frac{S_{\lambda_{k}}^{2}}{2\sigma_{\lambda_{k}}^{2}}\right\}\\ p\left(\sigma_{\lambda_{k}}^{2}\right)\propto {\left(\sigma_{\lambda_{k}}^{2}\right)}^{\Delta -1} \end{array}$$ (3)

where $\Delta =-\frac{\left(n_{\lambda_{k}}-1\right)}{2}$, such as $0<n_{\lambda_{k}}<1$ and, consequently, 0<Δ<1/2. Here $n_{\lambda_{k}}$ is a arbitrary value. In this study we assumed $n_{\lambda_{k}}=0.95$. Therefore, for $\sigma_{\lambda_{k}}^{2}$, *k* = 1, 2,…, 8, the scaled inverse chi-square prior is represented by $\upsilon_{\lambda_{k}}=\;-2\Delta$ and $S_{\lambda_{k}}^{2}=0$ as follows:

$$\sigma_{\lambda_{k}}^{2}\sim Inv-scaled-\chi^{2}(\upsilon_{\lambda_{k}},S_{\lambda_{k}}^{2})\to {\left(\sigma_{\lambda_{k}}^{2}\right)}^{\left(\Delta -1\right)}.$$

Therefore, the joint prior distribution is as follows:

$$\begin{array}{l} p(\theta )=p(\beta |\mu_{\beta },\sigma_{\beta }^{2})p(\delta |\mu_{\delta },\sigma_{\delta }^{2})p(\sigma_{\delta }^{2}|\upsilon_{\delta },S_{\delta }^{2})p(\sigma_{e}^{2}|\upsilon_{e},S_{e}^{2})\times \\ \prod_{k}^{t}\left[p(\lambda_{k}|\mu_{\lambda_{k}},\sigma_{\lambda_{k}}^{2})p(\sigma_{\lambda_{k}}^{2}|\upsilon_{\lambda_{k}},S_{\lambda_{k}}^{2})p\left(\alpha_{k}\right)p\left(\gamma_{k}\right)\right] \end{array}$$

where $\theta =(\beta ,\;\delta ,\;\lambda_{k},\;\alpha_{k},\;\gamma_{k},\;\sigma_{e}^{2},\sigma_{\delta }^{2},\;\sigma_{\lambda_{k}}^{2})$.

#### Likelihood and full conditional posteriors for the AMMI model parameters

The posterior probability distribution is a combination of the likelihood function (information from the data) and the prior probability distributions. The likelihood function for the AMMI model was implemented as follows:

$$L(\bar{\theta },\sigma_{e}^{2}|y)={\left(2\pi |I\sigma_{e}^{2}|\right)}^{-\frac{n}{2}}exp\left\{-\frac{1}{2\sigma_{e}^{2}}{\left(y-X_{1}\beta -Z\delta -\Theta \right)}^{\prime }\left(y-X_{1}\beta -Z\delta -\Theta \right)\right\}$$ (4)

where $\Theta =\sum_{k=1}^{t}\lambda_{k}diag(Z\alpha_{k})X_{2}\gamma_{k}$ and $\bar{\theta }=\{\beta ,\delta ,\lambda_{k}\alpha_{k}\gamma_{k}\}$.

Applying Bayes’ theorem gives the full posterior probability distribution as follows:

$$\begin{array}{l} p(\theta |y)\propto L(\bar{\theta }|y)p(\beta )p(\delta |\mu_{\delta },\sigma_{\delta }^{2})p(\sigma_{\delta }^{2}|\upsilon_{\delta },S_{\delta }^{2})p(\sigma_{e}^{2}|\upsilon_{e},S_{e}^{2})\times \\ \prod_{k}^{t}\left[p(\lambda_{k}|\mu_{\lambda_{k}},\sigma_{\lambda_{k}}^{2})p(\sigma_{\lambda_{k}}^{2}|\upsilon_{\lambda_{k}},S_{\lambda_{k}}^{2})p(\alpha_{k})p(\gamma_{k})\right] \end{array}$$ (5)

The full conditional distributions are as follows:

$$\begin{array}{l} p(\beta |\ldots )\propto exp\left\{-\frac{1}{2\sigma_{e}^{2}}{\left[A-(X_{1}´X_{1}){X^{\prime }}_{1}\beta \right]}^{\prime }\left({X^{\prime }}_{1}X_{1}\right)\left[A-({X^{\prime }}_{1}X_{1}){X^{\prime }}_{1}\beta \right]\right\}\\ p(\beta |\ldots )\sim N\left[{\left({X^{\prime }}_{1}X_{1}\right)}^{-1}{X^{\prime }}_{1}A,{\left({X^{\prime }}_{1}X_{1}\right)}^{-1}\sigma_{e}^{2}\right] \end{array}$$ (6)

where **A** = **y**-**Zδ**-**Θ** and *p* ( |…) is the conditional of all of the other parameters of the model.

$$\begin{array}{l} p(\delta |\ldots )\propto exp\;\left\{-\frac{1}{2\sigma_{e}^{2}}\;\left[\delta -\;\left(Z^{\prime }Z+I\frac{\sigma_{e}^{2}}{\sigma_{\delta }^{2}}\right)Z^{\prime }B\right]\text{'}{\left(Z^{\prime }Z+I\frac{\sigma_{e}^{2}}{\sigma_{\delta }^{2}}Z^{\prime }B\right)}^{-1}\left[\delta -\;\left(Z^{\prime }Z+I\frac{\sigma_{e}^{2}}{\sigma_{\delta }^{2}}\right)Z^{\prime }B\right]\right\}\\ p(\delta |\ldots )\sim N\left[{\left(Z^{\prime }Z+I\frac{\sigma_{e}^{2}}{\sigma_{\delta }^{2}}\right)}^{-1}B,{\left(Z^{\prime }Z+I\frac{\sigma_{e}^{2}}{\sigma_{\delta }^{2}}\right)}^{-1}\sigma_{e}^{2}\right] \end{array}$$ (7)

where **B** = **y**−**X**_1_ **β**−**Θ**.

$$\begin{array}{l} p(\sigma_{e}^{2}|\ldots )\propto {\left(\sigma_{e}^{2}\right)}^{-\left(\frac{n}{2}+1\right)}exp\left\{-\frac{1}{2\sigma_{e}^{2}}{\left(y-\theta \right)}^{\prime }\left(y-\theta \right)\right\}\\ p(\sigma_{e}^{2}|\ldots )\sim Inv-scaled-\chi^{2}\left(n,\left[{\left(y-\theta \right)}^{\prime }(y-\theta )\right]/n\right) \end{array}$$ (8)

$$\begin{array}{l} p(\sigma_{\delta }^{2}|\ldots )\propto {\left(\sigma_{\delta }^{2}\right)}^{-\left(\frac{n_{\delta }}{2}+1\right)}exp\left\{-\frac{1}{2\sigma_{\delta }^{2}}\left(\delta^{\prime }\delta \right)\right\}\\ \sigma_{\delta }^{2}|\ldots \sim Inv-scaled-\chi^{2}\left(n_{\delta },\left(\delta^{\prime }\delta \right)/n_{\delta }\right) \end{array}$$ (9)

$$\begin{array}{l} p(\lambda_{k}|\ldots )\propto exp\;\left\{\frac{1}{2\sigma_{e}^{2}}\;\left[\left(C-\phi \lambda_{k}\right)\text{'}\left(C-\phi \lambda_{k}\right)+(\lambda_{k}-\mu \lambda_{k})\text{'}\frac{\sigma_{e}^{2}}{\sigma_{\lambda_{k}}^{2}}(\lambda_{k}-\mu_{\lambda_{k}})\right]\;\right\}\\ p(\lambda_{k}|\ldots )\sim N^{+}\left[{\left(\phi^{\prime }\phi +\frac{\sigma_{e}^{2}}{\sigma_{\lambda_{k}}^{2}}\right)}^{-1}\phi^{\prime }C,{\left(\phi^{\prime }\phi +\frac{\sigma_{e}^{2}}{\sigma_{\lambda_{k}}^{2}}\right)}^{-1}\sigma_{e}^{2}\right] \end{array}$$ (10)

where **C** = **y**-**Xβ**-**Zδ**-**D** and *ϕ* = *diag*(**Zα**_*k*_)**X**_2_ **γ**_*k*_, for *λ*_1_ ≥ *λ*_2_,…,*λ*_*t*_ ≥ 0

In this formulation, the posterior mean of the singular value is similar to a ridge regression estimator. The posterior mean obtained by Crossa et al. [21] and Oliveira et al. [25] is similar to (*ϕ'ϕ*)^-1^ *ϕ'* **C**, referencing the least squares estimator. The distribution of the variance of the singular values is as follows:

$$\begin{array}{l} p(\sigma_{\lambda_{k}}^{2}|...)\propto \prod_{k=1}^{t}{\left(\sigma_{\lambda_{k}}^{2}\right)}^{\frac{-1}{2}}exp\left\{-\frac{1}{2\sigma_{\lambda_{k}}^{2}}(\lambda_{k}^{2})\right\}{\left(\sigma_{\lambda_{k}}^{2}\right)}^{\left(-1+\Delta \right)}\\ p(\sigma_{\lambda_{k}}^{2}|...)\propto \prod_{k=1}^{t}{\left(\sigma_{\lambda_{k}}^{2}\right)}^{\left[-\frac{\left(1-2\Delta \right)}{2}-1\right]}exp\left\{-\frac{\left(\lambda_{k}^{2}\right)}{2\sigma_{\lambda_{k}}^{2}}\frac{\left(1-2\Delta \right)}{\left(1-2\Delta \right)}\right\}\\ p(\sigma_{\lambda_{k}}^{2}|...)\propto \prod_{k=1}^{t}{\left(\sigma_{\lambda_{k}}^{2}\right)}^{\left[-\frac{\left(1-2\Delta \right)}{2}-1\right]}exp\left\{-\frac{\left(1-2\Delta \right)}{2\sigma_{\lambda_{k}}^{2}}\frac{\left(\lambda_{k}^{2}\right)}{\left(1-2\Delta \right)}\right\}\\ \sigma_{\lambda_{k}}^{2}|...\sim Inv-scaled-\chi^{2}\left(1-2\Delta ,\left[\lambda_{k}^{2}/(1-2\Delta )\right]\right) \end{array}$$ (11)

The posterior distribution for the singular vector are given by:

$$\begin{array}{l} p(\alpha_{k}|\ldots )\propto exp\left\{\frac{\lambda_{k}}{2\sigma_{e}^{2}}\left[\alpha_{k^{\prime }}diag\left(X_{2}\gamma_{k}\right)\prime Z^{\prime }\right]\left(y-X_{1}\beta -Z\delta -D\right)\right\}\\ p(\alpha_{k}|\ldots )\sim VMF\left\{\frac{\lambda_{k}}{2\sigma_{e}^{2}},(diag\left(X_{2}\gamma_{k}\right)\prime \;Z^{\prime })\left(y-X_{1}\beta -Z\delta -D\right)\right\} \end{array}$$ (12)

$$\begin{array}{l} p(\gamma_{k}|...)\propto exp\left\{\frac{\lambda_{k}}{2\sigma_{g}^{2}}\left[{\gamma^{\prime }}_{k}diag\left(Z\alpha_{k}\right)'{X^{\prime }}_{2}\right]\left(y-X_{1}\beta -Z\delta -D\right)\right\}\\ p(\gamma_{k}|...)\sim VMF\left\{\frac{\lambda_{k}}{2\sigma_{g}^{2}},[diagZ\left(\alpha_{k}\right)'{X^{\prime }}_{2}]\left(y-X_{1}\beta -Z\delta -D\right)\right\} \end{array}$$ (13)

where VMF is a Von Mises—Fisher distribution

Because the problem is orthogonally restricted, the singular vectors **α**_*k*_ and **γ**_*k*_ are sampled by linear transformation [21,25]. The sampling is conducted in the corrected subspace, where there are no restrictions. The values are then returned to the correct space in ℜ^*p*^, orthogonal to *t*-1, in the dimension space *p* (*p = a* or *p = g*, according to the vector).

Because the full conditional posterior distributions for the model parameters are known, direct sampling can be performed. The Gibbs sampler was used in the MCMC method. Chain convergence was assessed using the methods introduced by Raftery and Lewis [29] and Heidelberger and Welch [30].

To compare the Bayesian shrinkage AMMI method (AMMIBS) with traditional methods, four approaches were used. In the first approach, a classic AMMI analysis was performed using the analysis of variance (ANOVA). The number of components was selected using the F-test by Gollob [2], $F_{G}=\frac{\lambda_{k}^{2}/df_{\left(G\right)}}{S^{2}}$, and the F-test by Cornelius et al. [31],$F_{R}=\left(SQ(GE)-\sum_{k=1}^{t}\lambda_{k}^{2}\right)/df_{R}S^{2}$ , where *S* ^2^ is the joint mean squared error, *SQ*(*GE*) is the sum of the squares of the interaction, and *df*_(G)_ = *g*+*a*−1−2*k* and *df*_*R*_ = (*g*−1−*p*)(*a*−1−*p*) are the corresponding degrees of freedom. Both F-based tests were applied using a *α* = 0.05

In the second approach, a singular value decomposition of the BLUPs matrix for the GE interaction was performed using a mixed model with the genotype and interaction effects modeled as random. This is the same model represented by Eq 2, except the singular value decomposition is applied directly in the GE BLUPs using the model as follows:

$$y=\;X_{1}\beta +\;Z\delta +Wi+\epsilon$$ (14)

where **w** is the matrix of the random effect for GE interaction in classical mixed models. The estimates of the components of variance were obtained using expectation/maximization-restricted maximum likelihood (EM-REML) estimators. Vector **i**, which corresponds to the interactions, was transformed to the GE matrix, which was then used for the decomposition GE = ULV, where U and V are the singular vectors matrices for the genotypes and the environments, respectively, and L is the diagonal matrix of the singular values.

In the third approach, the AMMI analysis was subjected to the shrinkage estimator by Cornelius and Crossa [10] previously described. In the fourth approach, the Bayesian AMMI analysis (AMMIB) was performed as described in Crossa et al. [21] and Oliveira et al. [25].

#### Criterion to select Bayesian Shrinkage AMMI model

The use of a additional criterion to selection components could be not an important issue in Bayesian AMMI since all information about the parameter is available on its posterior distribution. Thus, if we use the *ad hoc* threshold proposed by Cornelius and Crossa [10] for AMMI shrinkage we can to select the AMMI model based on the hypothesis *H*_0_: *λ*_*k*_ ≤ 1. Therefore, if the *P*_*λ* = 1_ ≤ 0.05, where *P*_*λ* = 1_ is the percentile related to *λ* = 1 in the *p*(*λ*_*k*_|…) posterior distribution, we can retain the kth component or discard otherwise. Since this criterion uses a *ad hoc* value we propose others measures in order to exploit the advantages of shrinkage.

In this study we used an additional test based on Bayes Factor as a comparative measure where the choice of AMMI model (AMMI0, AMMI1…AMMI_k_) will be depend of the evidence e ≥ 12.79db where *e* = 10*log_10_(*BF*) and

$$BF=\left[\frac{p(\theta_{f}|y)}{p(\theta_{f-i}|y)}\right]$$

where *θ*_*f*_é a full model using *p* principal components and *p*(*θ*_*f-i*_) is the reduced model with *p-1* principal components where *i* = 1,2,3….*p*. The test was performed initially considering the score for high dimensional model and when e ≥ 12.79db the test was stopped [32]. This criterion consider a 0.95 of probability or 19:1 of chance to accept full model over the reduced one.

Additional comparison was accomplished using different cross-validation approach based on leave-one-out method proposed by [11] and [33]. Dias and Krzanowski [11] method is based on Prediction Sum Square (PRESS) criterion corrected by the number of degree of freedom retained on the *AMMI*_*k*_ and the number of degree of freedom remained after adjusting the *kth* component. This is justified since the PRESS in this method is monotonically decreasing and W statistics became necessary. The Gabriel [33] method uses only the PRESS criterion since its cross-validation approach present a "Ockham's profile" where the "pattern" and "noise" can be found by "Ockham's hill" in a plot using a Statistical efficiency criterion given by *SE* = *PRESS*_*full*_/*PRESS*_*k*_ where full related to raw data and *PRESS*_*k*_ to the prediction sum square related to kth component. The Gabriel method was used in both Bayesian and classical AMMI. However, in cross-validation in AMMI Bayesian methods we prefer to use only the inner product *λ*_*k*_ *α*_*ik*_ *γ*_*jk*_ and calculating the PRESS in order to gain computational efficiency and avoiding arbitrary choice of signal in singular vectors.

### Model parameter inference

The estimates for $\sigma_{e}^{2}$, $\sigma_{\delta }^{2}$, $\sigma_{\lambda_{k}}^{2}$ *λ*_*k*_, **δ**, and **β** were obtained using the MCMC samples means. The sample means for the singular vectors do not satisfy the restriction of orthonormality in the model. Therefore, the estimates for **α** and **γ** were obtained by orthonormalization of the matrices of the means $\bar{\alpha }$ and $\bar{\gamma }$, respectively, performed using the method proposed by Liu [24].

The univariate highest credible posterior density regions for the parameters were constructed using the method proposed by Chen and Shao [34], and implemented in the R statistical software [35].

The bivariate credible regions for the genotypic scores $\left(\alpha_{1}\sqrt{\lambda_{1}},\alpha_{2}\sqrt{\lambda_{2}}\right)$ and the environment scores $\left(\gamma_{1}\sqrt{\lambda_{1}},\gamma_{2}\sqrt{\lambda_{2}}\right)$ were constructed using the method in Ooms [36] and Oliveira et al. [25].

## Results

MCMC chains were simulated using 188,000 iterations for each parameter of the AMMIBS and AMMIB models. For each chain, the first 8,000 observations were discarded (*burn-in*) and values were saved at every 20^th^ observation (*thinning*), resulting in a sample size of 9,000. Convergence of the chains was evaluated using the criteria from Raftery and Lewis [29] and Heidelberger and Welch [30]. The results of the tests showed that all of the parameters had good convergence properties with a dependence factor that was always less than 5 (I<5) and that they all passed the stationarity test, indicating that convergence was achieved.

**Fig 1:**
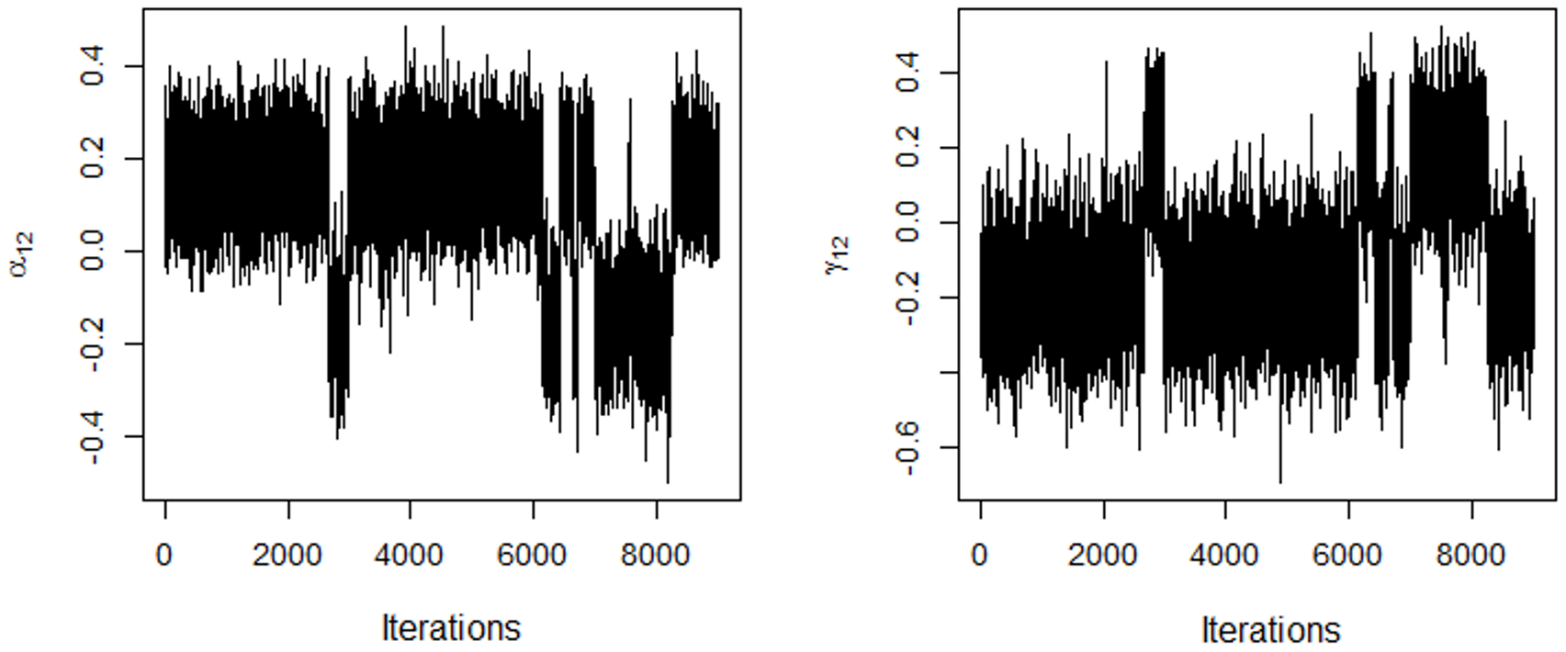
Traces of the chains for the first two coordinates of the genotype and environment singular vectors using the AMMIB model.

**Fig 2:**
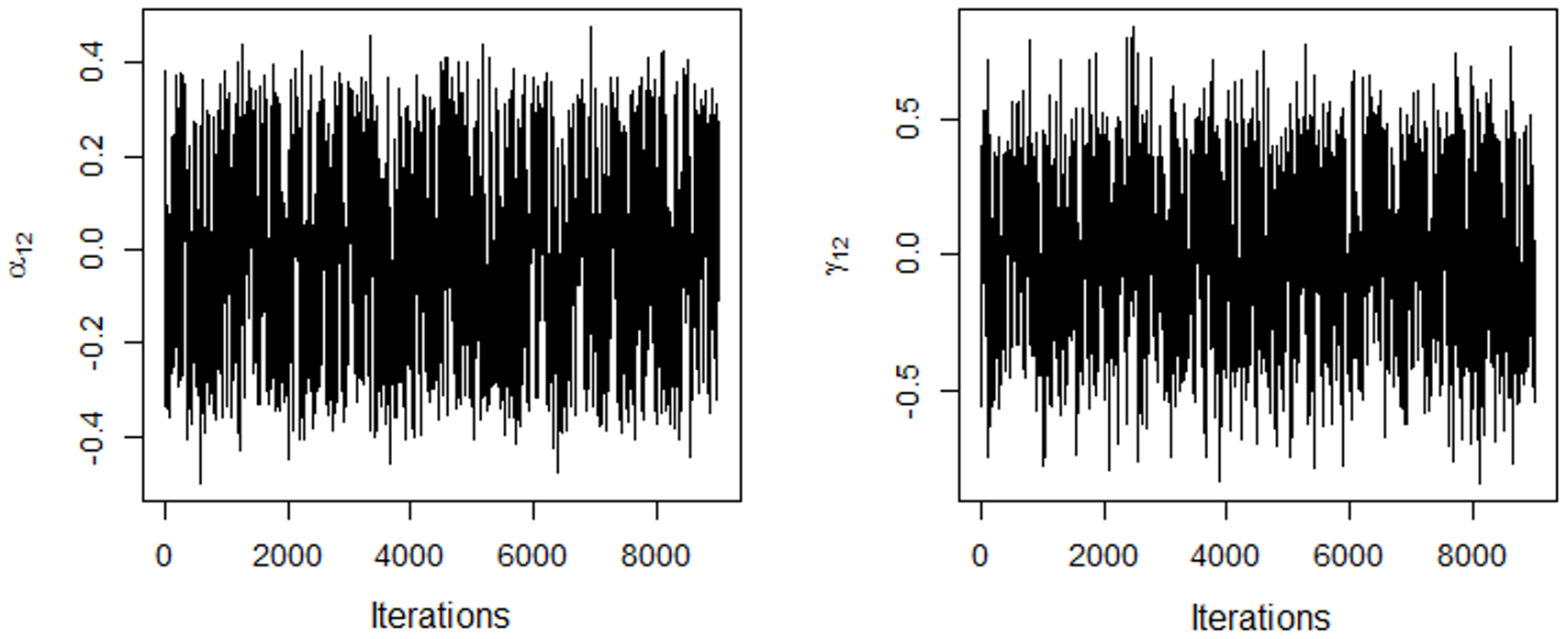
Traces of the chains for the first two coordinates of the genotype and environment singular vectors using the AMMIBS model, without restricting the solutions.

In the AMMIB model, the chains for the coordinates of the singular vectors (starting with the second genotype and environment vectors) converged to two solutions, equal in absolute value, similar to the results found by Oliveira et al. [25], with uninformative priors for the singular values. Fig 1 shows the traces for the first coordinates of the singular vectors associated with *λ*_2_ in the AMMIB model. The data show a change of the periodicity in the convergence that separates the chains into sign lags. This behavior was not apparent in the AMMIBS model (Fig 2). In both cases, the choice of sign is arbitrary.

**Fig 3:**
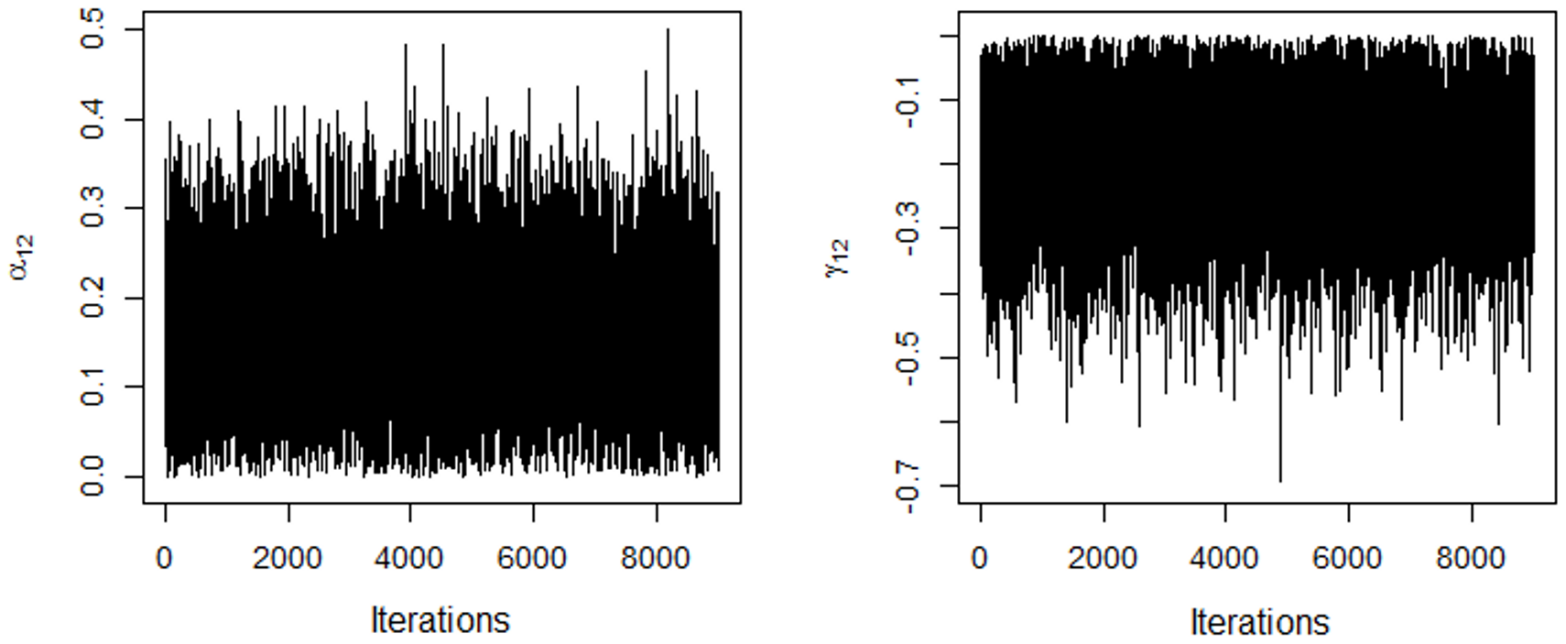
Traces of the chains for the first two coordinates of the genotype and environment singular vectors using the AMMIBS model, with restriction of sign for solutions.

The property of arbitrariness of the singular vectors sign is a characteristic of the principal component analysis. This characteristic ensures that the chains satisfy *E*[*α*_*ik*_] = 0 and *E*[*γ*_*jk*_] = 0 for *k* ≥ 2. One of the solutions (negative or positive) must be chosen; the choice does not affect the interpretation of the biplot nor the inner product. When the solution is chosen, the other terms adjust automatically. The solution with a positive sign for the first coordinate of each genotype singular vector was chosen. The signs of the other coordinates and the coordinates of the corresponding environment vector are then determined. This choice produced absolute singular values similar to those in Fig 1. Fig 3 shows the traces for the first coordinates of the second genotype and environment singular vectors, respectively, for the AMMIBS analysis. By choosing the positive solution, *α*_12_ and *γ*_12_ have opposite signs (see Fig 1).

This figure shows the negative association between genotype 1 and environment 1. Regardless of the sign chosen, *α*_12_ and *γ*_12_ have opposite signs (Fig 1).

**Fig 4:**
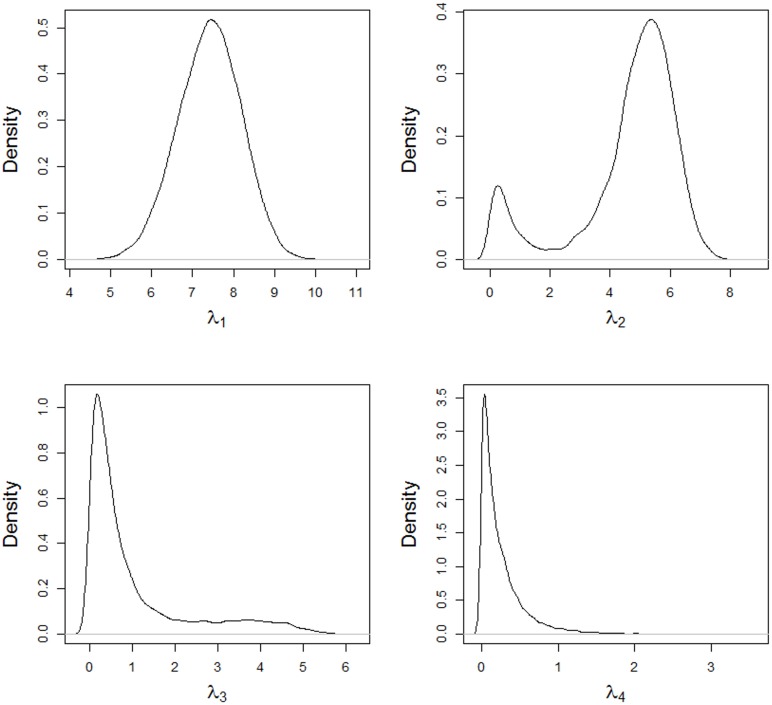
Posterior densities of the singular values *λ*_1_, *λ*_2_, *λ*_3_ and *λ*_4_, obtained using the AMMIBS model.

**Fig 5:**
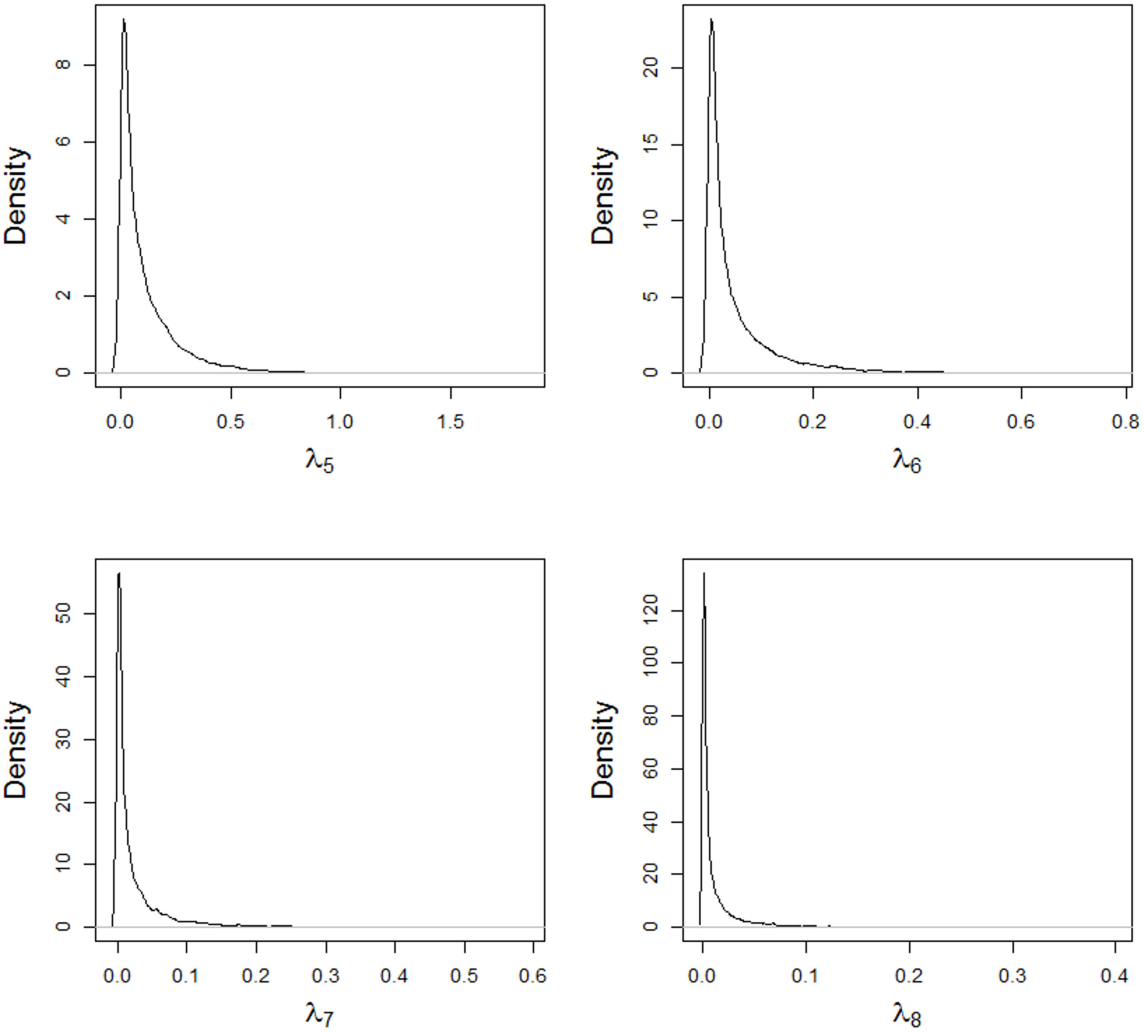
Posterior densities of the singular values *λ*_5_, *λ*_6_, *λ*_7_ and *λ*_8_, obtained using the AMMIBS model.

The posterior densities of the singular values for the AMMIBS model are shown in Figs 4 and 5. Starting in the third singular value, the chains converge to near zero, which shows a larger shrinkage when a specific variance is used in the truncated normal. Utilizing the prior for specific variance of the singular values means that the conditional posterior distribution for the second singular value is bimodal. This agrees with Ter Braak’s findings [37], when specific variances are assumed that have shrinkage effects on the parameters.

Because of this feature, the posterior mean for the second singular value was slightly shrunk, but the posterior mean was near zero for the others higher-dimensional singular values. On the other hand, the posterior of the second singular value presented values two modes being one of them lower than 1 (Cornelius and Crossa *ad hoc* criterion for classical AMMI shrinkage).

**Fig 6:**
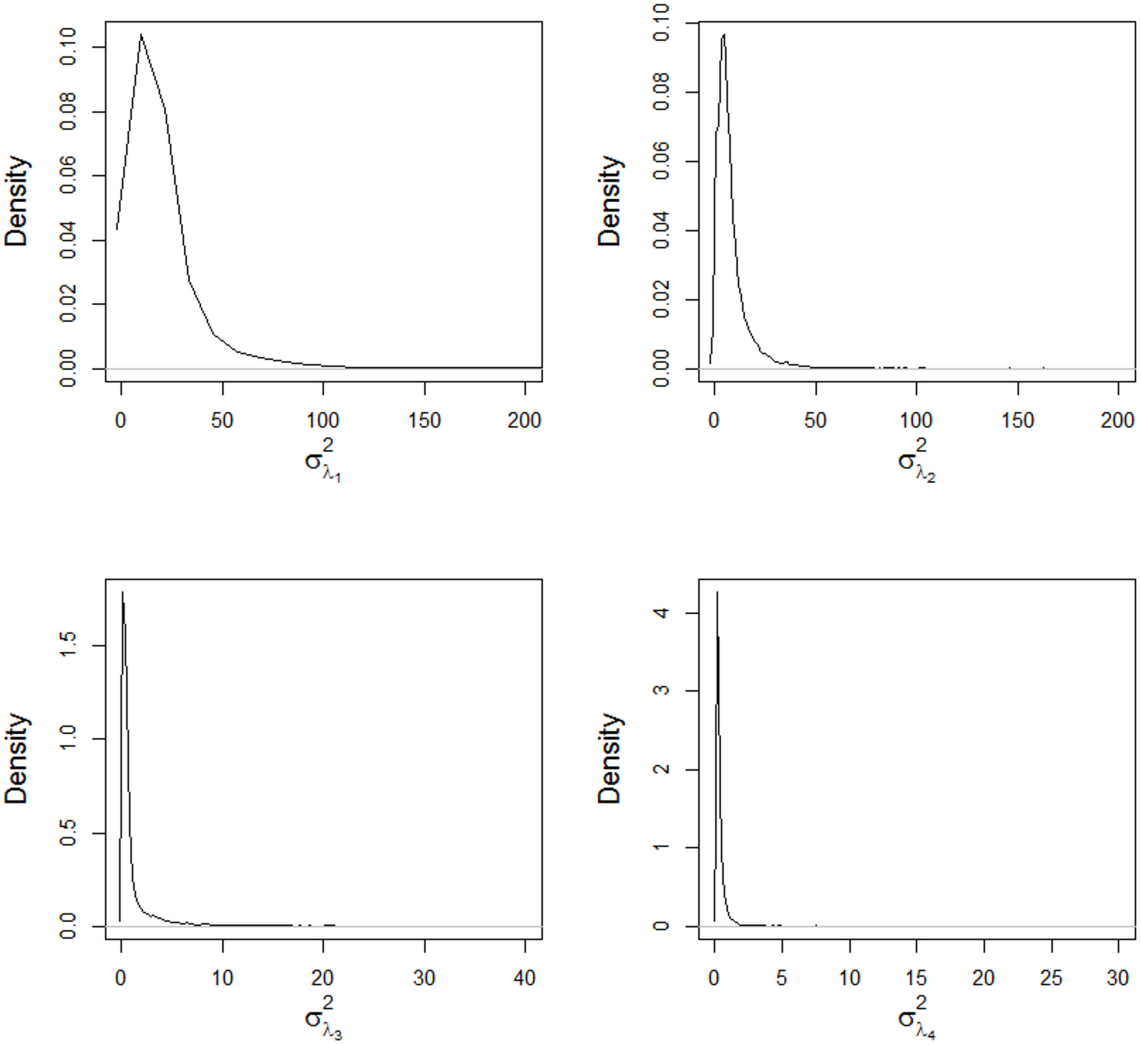
Posterior densities of the variance components for the singular values $\sigma_{\lambda_{1}}^{2},$ $\sigma_{\lambda_{2}}^{2},$ $\sigma_{\lambda_{3}}^{2}$ and $\sigma_{\lambda_{4}}^{2}$, obtained using the AMMIBS model.

**Fig 7:**
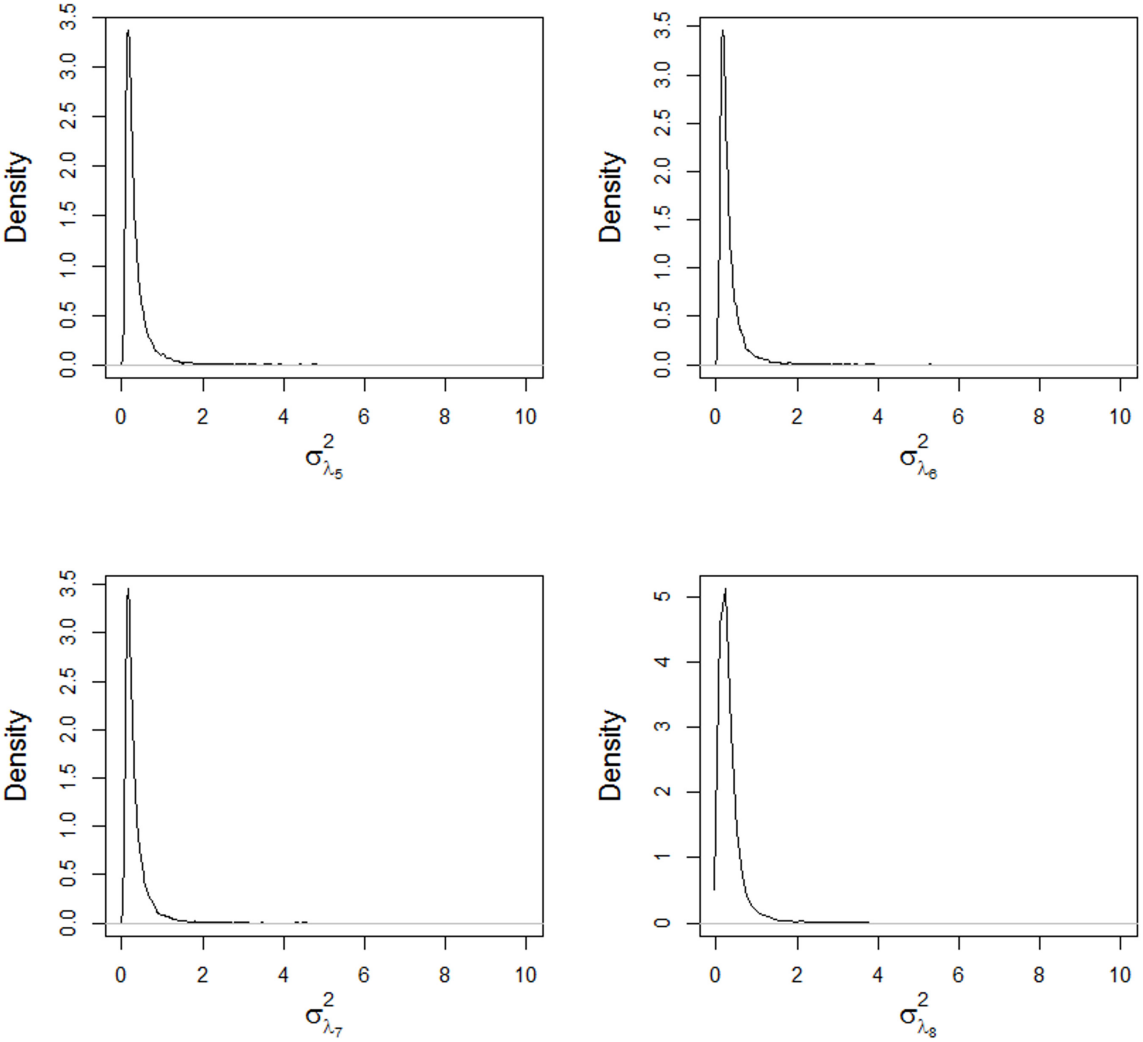
Posterior densities of the variance components for the singular values $\sigma_{\lambda_{5}}^{2},$ $\sigma_{\lambda_{6}}^{2},$ $\sigma_{\lambda_{7}}^{2}$ and $\sigma_{\lambda_{8}}^{2}$, obtained using the AMMIBS model.

The posterior densities of the components of variance for the singular values are shown in Figs 6 and 7. Only the first two components have high means and variances, while the other components have values near zero with low variance, resulting in shrinkage of the estimates of the singular values (Figs 4 and 5). The shrinkage effect on the estimates of the singular values are directly associated with the components of the variance, as shown in Eq 14. The mean values for these components are shown in Table 2 with their corresponding 95% credible intervals.

**Table 2:** Posterior mean estimates and the 95% credible interval (CI) for the variance components of the singular values.

Parameter	Mean	Sd	Lim. Inf.	Lim. Sup.
$\sigma_{\lambda_{1}}^{2}$	19.524	68.699	1.977	51.057
$\sigma_{\lambda_{2}}^{2}$	8.226	12.467	0.046	24.110
$\sigma_{\lambda_{3}}^{2}$	1.232	3.451	0.052	5.062
$\sigma_{\lambda_{4}}^{2}$	0.402	1.056	0.050	1.047
$\sigma_{\lambda_{5}}^{2}$	0.342	0.392	0.049	0.927
$\sigma_{\lambda_{6}}^{2}$	0.332	0.379	0.049	0.879
$\sigma_{\lambda_{7}}^{2}$	0.334	0.367	0.049	0.890
$\sigma_{\lambda_{8}}^{2}$	0.339	0.822	0.046	0.887

### Point estimates and credible regions for the other model parameters

**Fig 8:**
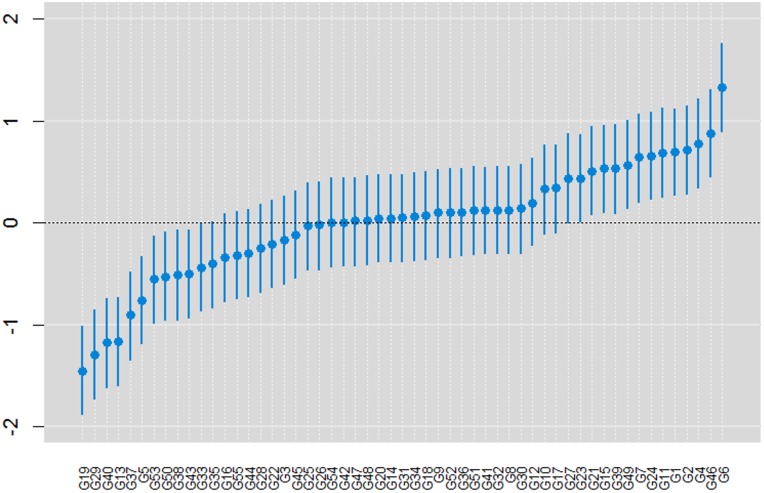
Posterior means and the 95% HPD intervals for the genotype effects.

The means of the components of variance, $\sigma_{\delta }^{2}$ and $\sigma_{e}^{2}$, were 0.373 and 1.285 with a 95% highest posterior density (HPD) interval of [0.2262, 0.5426] and [1.1680, 1.406], respectively. The 95% HPD intervals for the primary genotype effects are shown in Fig 8 in increasing order. This ranking allows the genotypes that contribute most to the population mean to be identified, i.e., those that have only positive values in the HPD region (located to the right of genotype G17 in the Fig 8).

To make more accurate conclusions concerning the selection and recommendation of the best genotypes, the effects of the GE interaction must also be considered by analyzing the terms that describe it.

The posterior means for the components of the variance for the singular values that are responsible for shrinkage effects are shown in Table 2. These estimates are calculated using the AMMIBS model. The estimates of the singular values using the AMMIBS model, the AMMIB model, the BLUPs, the OLS and the shrunken estimates (*Shrink*.), proposed by Cornelius and Crossa [10,13], are shown in Table 3.

**Table 3:** Estimates of the singular values *λ*_*k*_ for the AMMI model using the AMMIBS model, the AMMIB model and the AMMI model with fixed and random GE (OLS and BLUPs) and the AMMI shrinkage developed by Cornelius.

Parameter	*AMMIBS*	*AMMIB*	*BLUPs*	*OLS*	*Shrink*.
*λ*_1_	7.411^§^	7.902^§^	7.027	9.559* ^+^ ^€^	6.652
*λ*_2_	4.589^§^	5.978^§^	5.887	7.580* ^+^	6.442
*λ*_3_	1.015	4.605^§^	5.278	6.156^+^	6.442
*λ*_4_	0.254	2.268^§^	4.309	6.007^+^	6.442
*λ*_5_	0.108	1.058	4.161	5.674^+^	2.783
*λ*_6_	0.051	0.478	3.857	3.920	0
*λ*_7_	0.025	0.213	2.766	3.306	0
*λ*_8_	0.013	0.109	2.379	<0.000	0

*Significant by the Cornelius F-test at a 0.05 significance level

^+^Significant by the Gollob F-test at a 0.05 significance level.

^€^Number of terms retained on the AMMI model based on leave-one-out cross validation using W's score by Dias and Krzanowski.

^§^ Model selection based on Bayes Factor.

The data in Table 3 show that the singular values in the AMMIB model from Crossa et al. [21] are slightly shrunken compared to the classical AMMI with GE, estimated by OLS or the mixed models (EBLUP). In the AMMIB model, the first five singular values have means above zero, but, by Bayesian Factor criterion AMMI4 model be chosen. This result is very similar to the values obtained by shrinkage AMMI from Cornelius and Crossa [10], and also by the Gollob F-test, which selected only five principal components. This result suggests that AMMIB tends to select models similar to the Cornelius and Crossa [10] shrinkage estimator, and these results are consistent with the test performed on the traditional AMMI model using the Gollob F-test.

The data in Table 3 also show that the AMMIBS model has a more pronounced shrinkage effect than the AMMIB model or the Cornelius shrinkage model. Specifically, the AMMIBS model captured 89.11% of the interactions explained by the model (not necessarily the raw interactions), while the AMMIB model captured 61.34% in the first two axes. The first two singular values from both models were very similar, suggesting that the AMMIBS model shrinks only the low-magnitude singular values, preserving the estimates of the first ones, capturing most of the interaction pattern than noise. The Bayesian shrinkage method proved to be the most parsimonious of the all methods, capturing most of the interactions with the lowest number of multiplicative terms to explain the interaction pattern.

The estimates of the first singular value from the AMMIBS, the AMMIB and the EBLUP models were similar to one another, but different from those obtained with the traditional AMMI and Cornelius estimators. Based on this result, the GE interaction matrix, obtained from the inner product of these methods, showed a pronounced shrinkage and, therefore, was similar to modeling the interaction as random in a mixed model. The AMMIB model estimates were also more similar to the AMMI model with EBLUP than to the AMMIBS model.

The model selected by the singular value posterior distribution in AMMBS was AMMI1. It was evident in Fig 3, since the threshold suggested by Cornelius and Crossa presented *p*_*λ* = 1_ ≤ 0.05 To be more exact, all values obtained in MCMC for the first singular value was higher than 1 for the first singular value. This results agree with Dias and Krzanowski [11] criterion where the best model was AMMI1 (Table 3). On the other hand, the BF criterion applied in Bayesian analysis suggests AMMI2 for AMMIBS and AMMI4 for AMMIB. The result for AMMIBS is similar to than obtained using Cornelius F-based test. Using classical AMMI analysis (OLS), the selected models were AMMI1, AMMI2 for Dias and Krzanowski [11] criterion and Cornelius F-based test respectively and AMMI5 for F-Gollob and Cornelius and Crossa shrinkage analysis.

**Fig 9:**
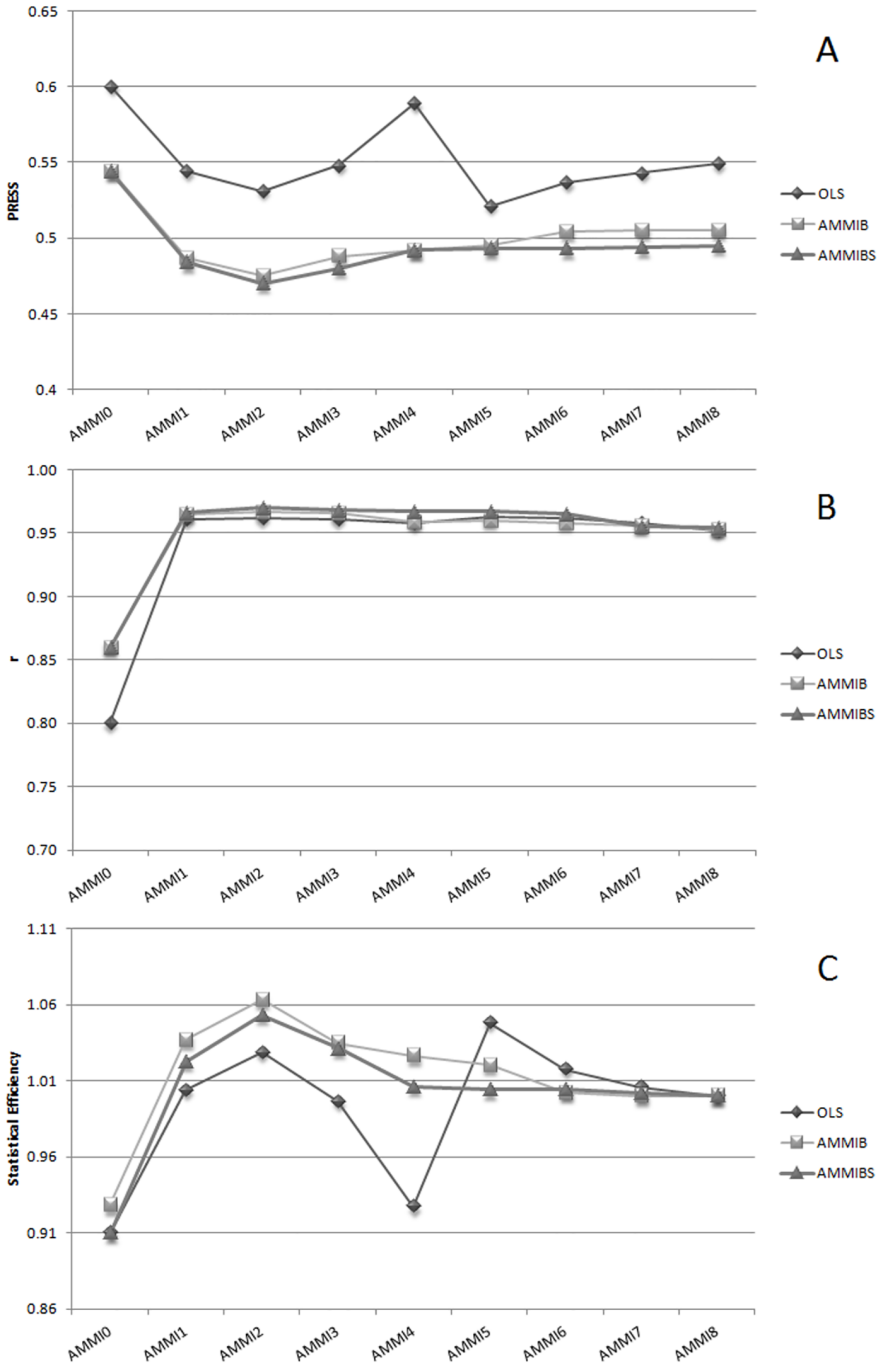
Ockham's plot related to classical AMMI model (OLS), Bayesian AMMI (AMMIB) and Bayesian Shrinkage AMMI (AMMIBS). (A) AMMI vs prediction sum square- PRESS (lowest is best—Ockham's valley); (B) AMMI vs correlation (r) between "true" and predicted cell values in GEI matrix; (C) AMMI vs Statistical Efficiency (higher is best—Ockham's hill).

In general the select models across different analysis were (AMMI1, AMMI2, AMMI4 and AMMI5). These divergent results can be explained when we analyze the Fig 9. In this figure it is evident that AMMI2 and AMMI5 presented two peak in the plot for statistical efficiency; where in OLS analysis the "Ockham's hill" was observed in AMMI5. But this model retain two times more degree of freedom than AMMI2. In both Bayesian analysis AMMI5 was marginal and the "Ockham's hill" was allocated at AMMI2 suggesting Bayesian methods the best approach to retain pattern and discarding noise using lesser degree of freedom. The predictive accuracy based on correlation for all models were equivalent showing marginal gain starting from AMMI1.

### Bivariate credible regions for the genotype and the environment scores

Bivariate credible regions were constructed for the AMMI2 biplot, similar to those presented by Oliveira et al. [25]. Genotypes or environments whose credible regions for the scores in the biplot include the origin are considered stable. In addition, large overlaps between the credible regions indicate that the respective genotypes or environments have similar GE interactions.

**Fig 10:**
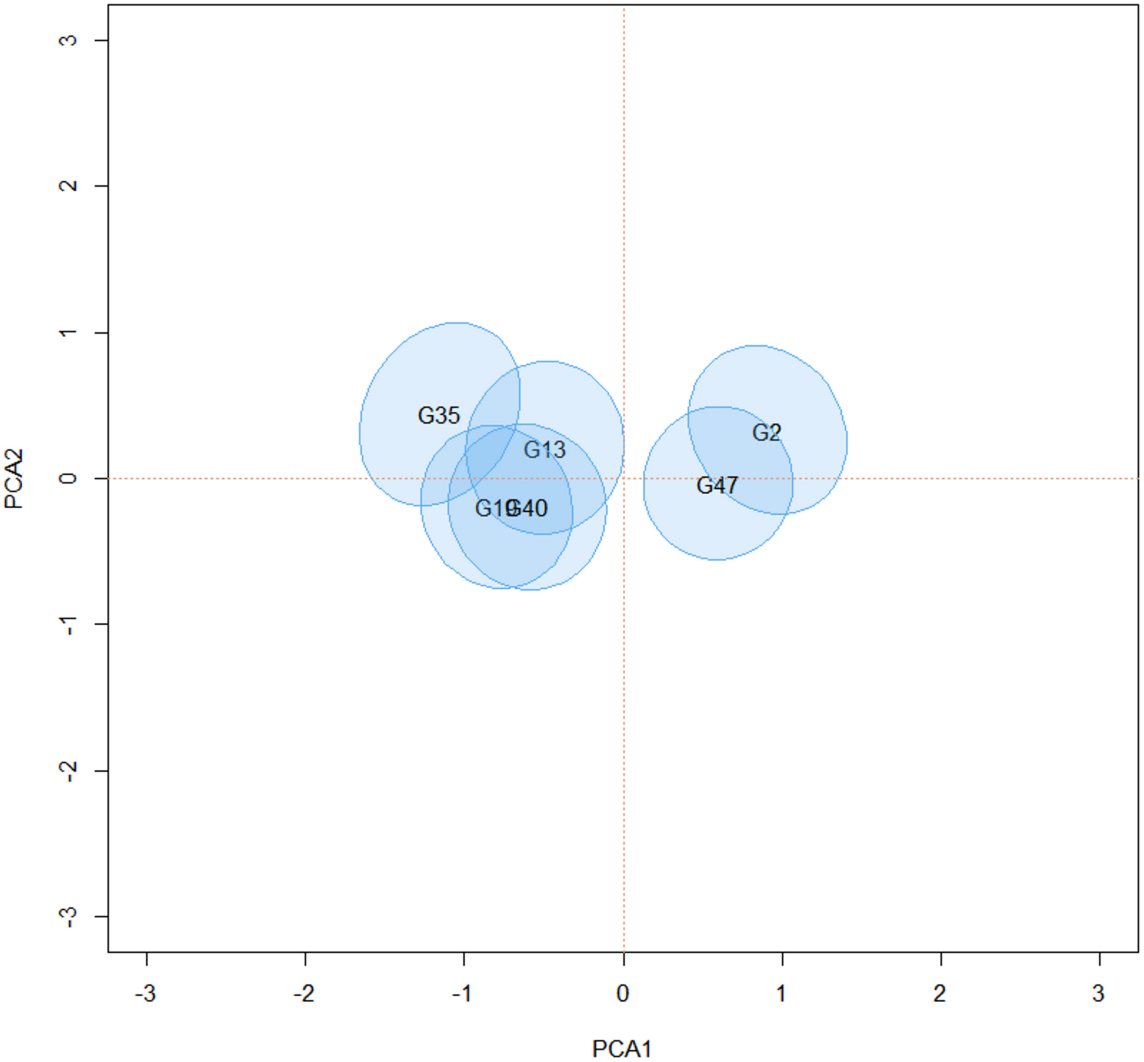
95% credible regions that do not include the origin for the first two principal axes of the genotype scores using the AMMIB analysis.

**Fig 11:**
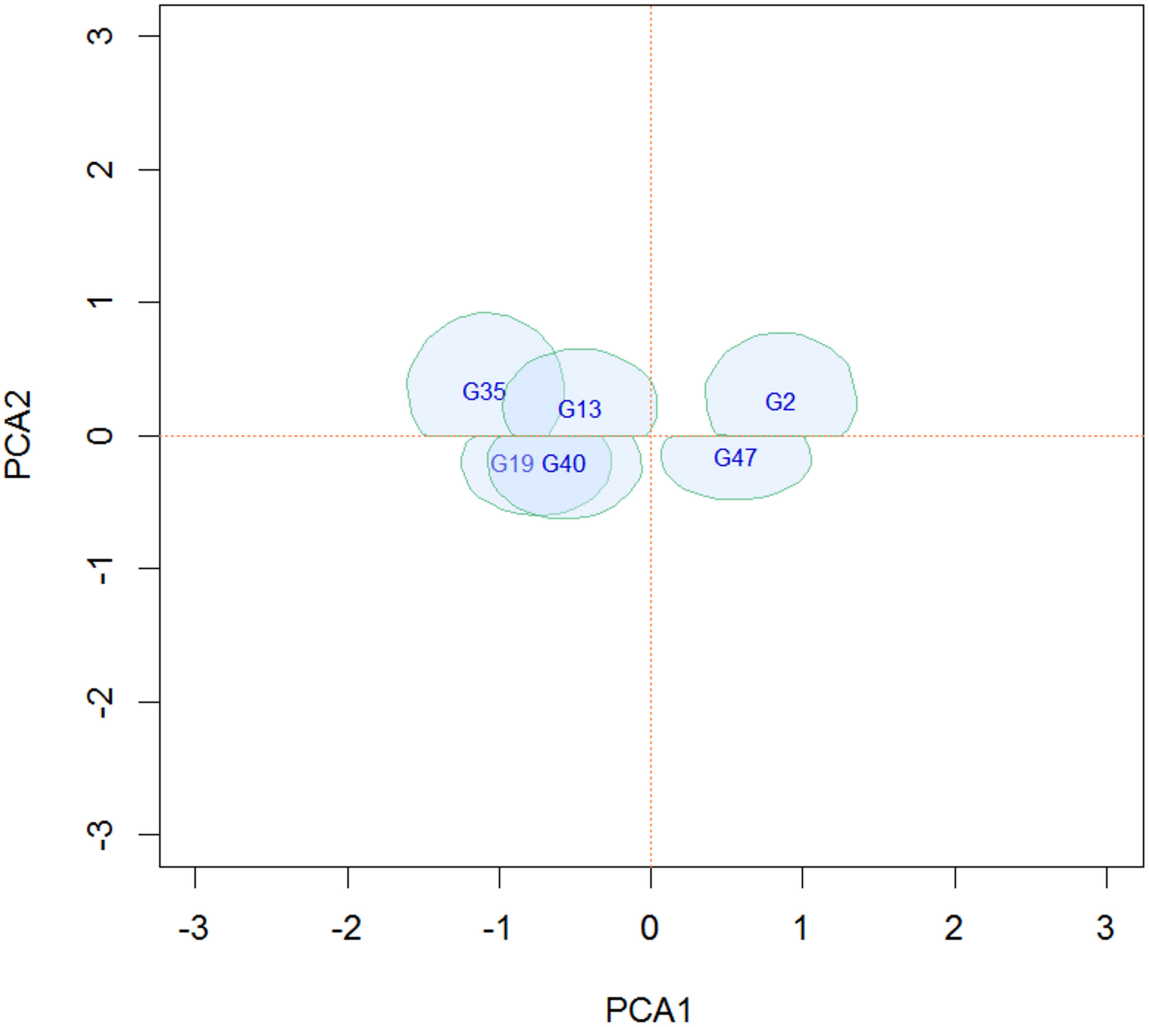
95% credible regions that do not include the origin for the first two principal axes of the genotype scores using the AMMIBS analysis.

The Figs 10 and 11 show the same groups of genotypes for the AMMIB and the AMMIBS analyses, respectively. The figures show that the grouping pattern is retained, even with the truncation of solutions of the second singular vectors in the AMMIBS analysis.

The genotypes shown in Fig 11 have nonzero values for the interactions (unstable genotypes), and two homogeneous groups can be formed, i.e., one group comprising G2 and G47 and the other group comprising G13, G19, G35 and G40. These two groups are responsible for many of the significant contribution to the GE interaction. Only the regions that do not include the origin are shown in the figure for simplicity and ease of interpretation. The bivariate credible regions in this biplot were not regular in the second axis. This distortion was from the truncation in the choice of the sign that, together with the shrinkage model, prevented the correct selection of the points in the chain of singular vectors (see Fig 2). This problem did not occur in the AMMIB model and the separation of the chain into lags and the choice of sign were more pronounced (Fig 1). In Fig 11, the change in the signs during the MCMC is in evidence, but the data show that the absolute value of the two estimates and the position of the bivariate credible regions in the biplot for the two methods did not differ.

**Fig 12:**
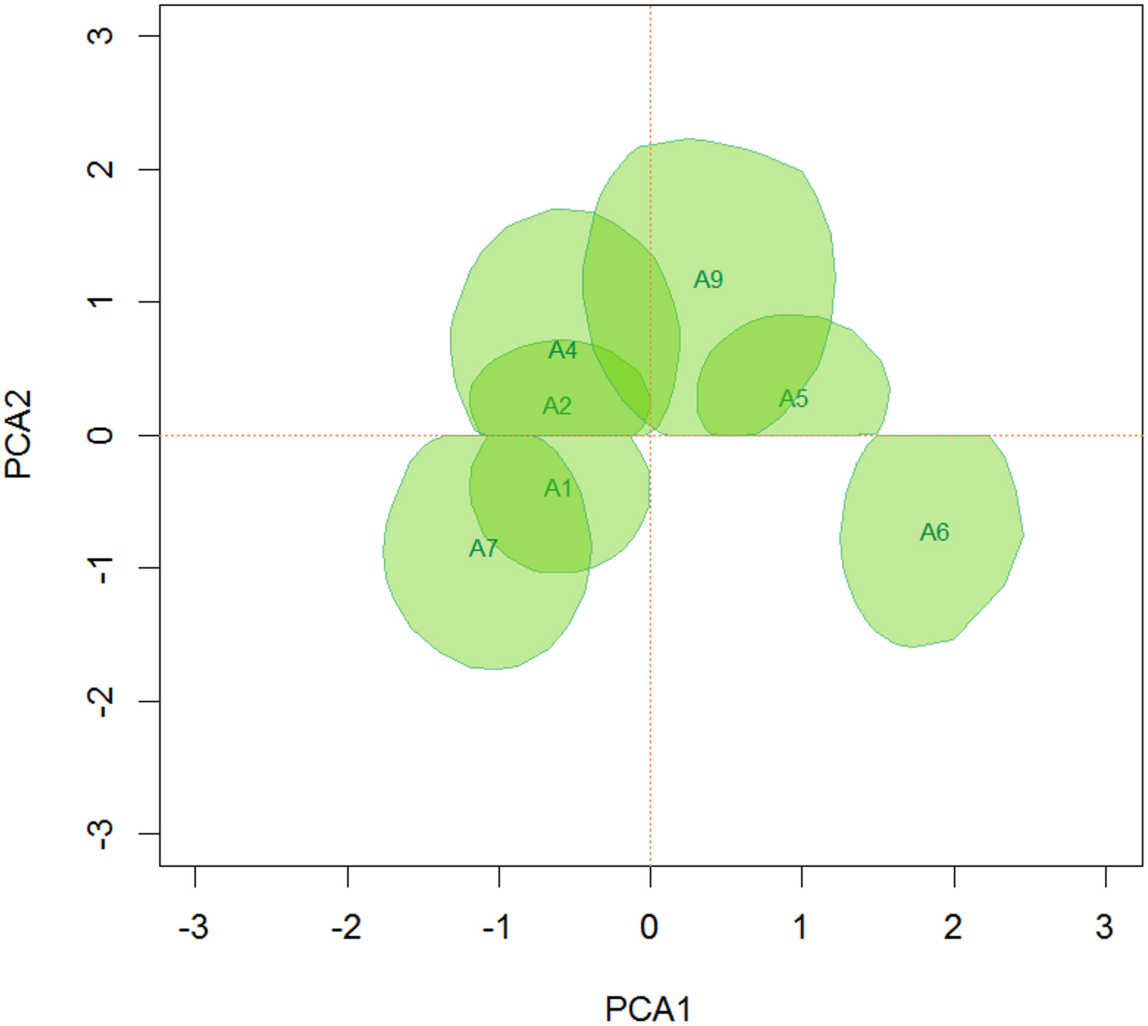
95% credible regions that do not include the origin for the first two principal axis of the environmental scores.

The same procedure was performed to create homogeneous groups of environments with respect to the interaction effect. Similar to the genotypes, the results for environments A3 and A8 are not shown in the biplot for simplicity because they do not contribute significantly to the interaction effect. The others were classified by similarity among groups, resulting in the three groups shown in Fig 12, i.e., (A6), (A1 and A7) and (A2, A4, A5 and A9). In this case, the same restriction regarding the truncation when choosing the sign was applied.

**Fig 13:**
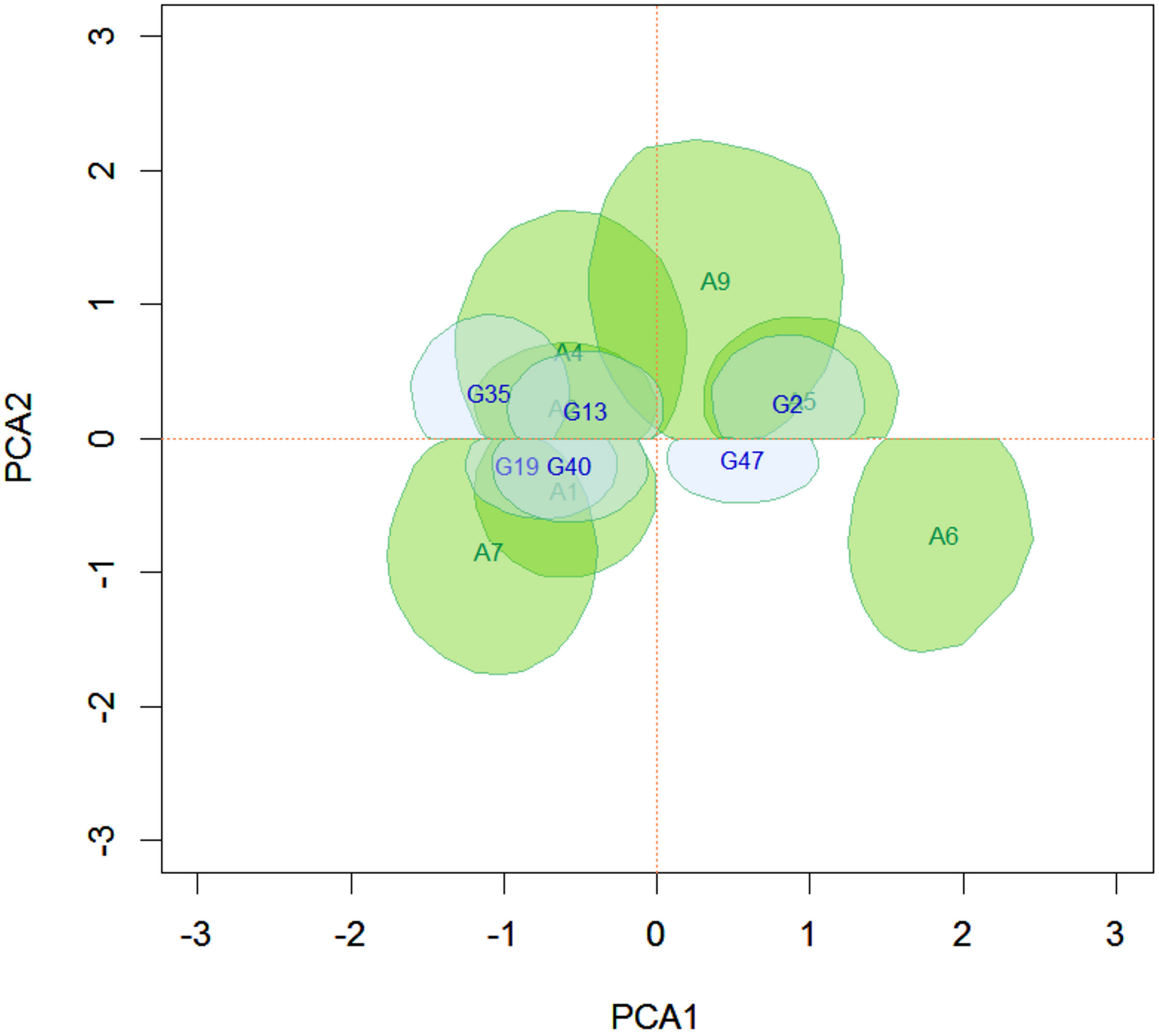
95% credible regions that do not include the origin for the first two principal axes of the genotype and the environmental scores.

Using the biplot, the adaptability between genotypes and environments was also analyzed (Fig 13). The superposition of the credible regions showed that the group consisting of G13, G19, G35 and G40 was specifically adapted to the environment group consisting of A1, A2, A4 and A7, while genotype G2, based on the overlap of the credible regions, appears to be specifically adapted to environments A2 and A9. Genotype G47 does not overlap the environments because of the restriction of the solution of the second singular vector; however, the proximity of the credible regions in the truncated area indicates that G47 would be adapted to environments A2 and A9. Apparently, none of the genotypes would be adapted to environment A6.

These results, the univariate HPD regions for the BLUPs of the effects and the bivariate credible regions for the genotype and environment scores in the biplot can help breeders to select the best genotypes, providing statistical support for their choices.

## Discussion

In Bayesian inference, the number of bilinear components to be retained in AMMI model is selected using the Bayesian information criterion (BIC) and the Bayes factor (BF) [22, 24]. However, Cornelius and Crossa [10,13] showed that when shrinkage estimators are used, the zero singular values do not provide any information on interaction effects and can thus be excluded from the model. Therefore, by assigning specific variance to the singular values, the selection of the interaction terms is similar to a shrinkage estimator of fixed effects, i.e., singular values that contribute little or no information on the interaction pattern have estimates close to zero and are not included in the model, and those with true importance for the bilinear model have little or no shrinkage effect and are thus retained in the model.

The results of Cornelius and Crossa [10,13] showed that shrunken estimates for linear-bilinear models with fixed effects are superior to OLS and as good as or better than estimates produced by BLUPs for models with random effects. Their method, however, was subject to the limitations of models with fixed effects and, therefore, could only be used with balanced data and homogeneous variances. Another disadvantage is the need for extra steps in the iterative estimation process because the estimates obtained by the iterative method could violate the restrictions imposed on singular values, as observed in this study, where the restriction *λ*_*k*_ ≥ *λ*_*k*+1_ was generally not obeyed. To circumvent this violation of the model, it is assumed that *λ*_*k*_ = *λ*_*k*+1_ and a joint estimate for the terms violating this restriction is calculated, as followed in the present analyses (Table 3).

Our approach showed that shrinkage estimators can be obtained for singular values without violating the restrictions of the analysis allowing to obtain credible intervals for the principal components and, simultaneously, to select the best model, i.e., the one with the fewest components. Our approach is a Bayesian interpretation of the estimators developed by Cornelius and Crossa [10], although in practical terms, the selected model was more parsimonious (AMMI2 vs. AMMI5 and AMMI1 vs. AMMI5). Despite this difference, our method based on Bayes Factor or evidence criterion [32] converged to the same model indicated by the Cornelius F-test and purely Bayesian inference selected values similar to Dias and Krzanowski cross validation criterion. The first principal component showed no shrinkage compared with those obtained by the AMMIB and the AMMI models using a random GE matrix. This result showed that the model successfully retains axis with greater ability to capture the pattern and eliminates axis with high noise as confirmed by cross validation result.

In AMMIBS, the use of any test on the number of components might not be necessary since all information we need is present at posterior distribution. In this context, the model to be selected by the AMMIBS was AMMI1 instead AMMI 2 as by BF measure. This can be observed by the use of the posterior percentile in the Fig 1. However, this approach might be ambiguous since the second singular value present a high posterior average and hold the "Ockham hill" in the plot and, therefore, might be important to explain GEI. In this sense, we adopted a extra criterion by used of Bayes Factor. The Bayes Factor reveled the best model in a very parsimonious models (AMMI2) and in this context what is the best approach? A general and definitive answer is very difficult but in the Fig 9 we can obtain a clarification for the range of models observed. The AMMI OLS gives us a ambiguous response about the best model since AMMI5 and AMMI2 presenting two peaks being the Ockham´s hill localized at AMMI5. By the classical interpretation this could be the best model since it retain more pattern than noise. But the degree of freedom requested to adjust AMMI5 is two times larger than requested by AMMI2 and thus the best model could be AMMI2 in OLS context [11]. This reason was used by [11] in their approach to find the more parsimonious model by account for the number of degree of freedom used in PRESS adjustment. On the other hand, in Bayesian cross validation context, this problem was not observed since higher dimensional components were shrunk and no more than one peak was observed in Ockham's plot. Thus, the Bayes Factor was a good criterion to select model since all pattern was retained in the two first components—presenting lower PRESS than AMMI5-OLS. However, the Bayes Factor analysis presented a high computational cost because the number of rounds requested to obtain the criterion was almost equal to the number of components in AMMI model. In addition, in AMMIB, the BF criterion selected the worst AMMI model considering the predictive ability (AMMI4).

As alternative we applied a direct cutoff criterion using the threshold given by Cornelius and Crossa [10] for shrinkage AMMI on the posterior distribution. In this sense, the AMMI1 model was selected. It is obvious that AMMI 1 is not the best model as showed in Ockham's plot, but it is the second one. Therefore, we might claim the parsimonious principle and verifying that the number of replications requested by full model to obtain the same root mean square predictive difference (RMSPD) of AMMI1 and AMMI2 is 7.4 and 8 respectably. Thus, the loss of GEI pattern between choosing AMMI1 instead AMMI 2 could be marginal, but computably more efficient and therefore we suggested the AMMI1 model as the best model for our dataset. In addition, the direct posterior analysis as proposed here do not request several rounds of tests as in BF test and do not claim for Gaussian assumption about principal components as in F test—about this last claim, our result showed that Gaussian assumption, in general, is very strong.

It worth to highlight also that our BF algorithm was not optimized since we started by full model instead lower dimensional models. Thus, we suggest the development of a more efficient algorithms starting from more parsimonious models first in order to avoid more cycles of Bayes Factor test.

A good discussion about parsimony and predictive ability in AMMI models was given by Dias and Krzanowski [11]. These authors observed that cross-validation based on leave-one-out methods usually select more parsimonious models than F-test or cross-validation based on randomized methods. In other words, they found that leave-one-out selected AMMI1 while AMMI3 and AMMI2 were selected by F-Gollob and Cornelius F-test respectively. In addition these authors observed very larger range of different models selected by F-based tests and cross-validation as observed in this study, pointing the difficult task of selecting models in AMMI methods. In general, the authors concluded that cross-validation methods are more stable across several trials, more parsimonious than F-based tests and are free from Gaussian assumption.

Although it is very difficult to relate analytical proof for our empirical result, the relation between AMMIBS and F-based test is very similar. It is worth highlighting that different breeder's aims could be related to the number of components retained in the AMMI model and that predictive ability sometimes may be useful to breeder, but sometimes not [38]. In this work, we prefer to emphasize the advantage of applying models selection plus biplot inference using just a model and further discussion could be raised in a more practical study.

The primary difficulty encountered in the shrinkage estimators was in the separation of the sign of the singular vector obtained by the AMMIB method arising from the precision parameter of the von Mises-Fisher conditional distribution. Because the ratio of singular value and residual variance represents the concentration parameter, in high-dimensional coordinates, when the singular value is strongly shrunken, these vectors are sampled with high dispersion distribution or nearly uniformly over the hypersphere. Therefore, this effect combined with the arbitrary change in the sign showed that the separation of the chain into lags in high dimensional parameter is impractical.

Another cause of the lack of periodicity in the chain is from the Jeffreys priors for the specific variances, resulting in scaled inverse chi-square conditionals with only one degree of freedom of high-variance and improper posteriors for the singular values. To solve this situation, an extended prior, from Ter Braak et al. [28], was implemented to obtain a proper posterior distribution. However, as shown by Ter Braak et al. [28] and verified by the data in this study, the conditional posterior density can be bimodal, with one mode peaking near zero. According to Ter Braak [37], this is caused by the shrinkage effect provided by the prior. The posterior mean for the second singular value, also shown by Ter Braak [37], is the least squares estimate of this value times its shrinkage factor.

It was empirically shown that the absolute value of each coordinate is approximately equal to that obtained from the conventional analysis, and, therefore, this pattern was used to choose the sign (Figs 1 and 2).

The decision to use the absolute value of the chain resulted in point estimates of the singular values similar in the AMMI, the AMMIB, and the AMMIBS methods, but hindered the construction of the credible intervals. However, when there was little shrinkage of the singular value, the bivariate credible regions were not truncated, as observed for the first principal component. This implies that in models with a large amount of shrinkage, the inferences for the intervals can be hindered, even though the estimates of the posterior means are similar to conventional estimators.

Despite this limitation, overall, the AMMIBS model successfully chose the terms for the AMMI model and parameter estimates, and offers several benefits compared with traditional methods. For example, although not addressed in this study, the model can have heterogeneous variance in the data, which, as previously mentioned, would be a limitation of frequentist approaches; although some works have tried to address heterogeneity by weighted models [39]. Moreover, additional information, such as relatedness and historical experimental data can be incorporated with relative ease into the model. Although these advantages can also be obtained from mixed models in MET in versions equivalent to AMMI models and genotype main effect plus genotype environment effect (GGE) biplots [18,19,20], parametric confidence regions for the scores in the biplot are not easily incorporated [17,21]. Some suboptimal solutions have been proposed by Yang et al. [17], suggesting that confidence regions in the biplot be constructed using bootstrap resampling techniques. This method has been criticized by destroying the interaction pattern [16]. The critics emphasized that the position of the scores in a biplot is important for the analysis and mutually defined, so that the signs and values lose meaning when genotype scores are randomly separated from environment scores in the resampling process. But, in general, our results showed that Yang et al. [17] claim about biplot interpretation must be taking into account since the uncertain about relative position of environments and genotypes in the biplot may conduct to erroneous classification of stability and adaptability plus mega-environments discovery.

When estimating the singular values for the AMMIB model, the estimates were shrunk relative to the least squares solutions, which is consistent with the results in Crossa et al. [21] and Oliveira et al. [25]. However, as shown in Table 3, AMMIBS produced even more shrunken estimates, i.e., the results suggest that shrinkage using specific variance for the singular values was stronger for the high-dimensional terms and had little effect on the first two singular values. This implies that the AMMIBS model was more parsimonious because it retained only one non zero singular value and two principal components to describe the GE interaction. As described above, the inner product of these components produces shrunken GE estimators, showing that the AMMIBS model produces more shrunken interaction estimates than BLUPs from a mixed model, suggesting that the interaction is a random. The AMMIB model produced the same result, with an inner product much closer to the BLUP, suggesting that this model restricted by priors with a fixed estimator produces shrunken GE estimates that are similar to the BLUPs. It is worth to highlight that the BLUP method instead of shrinking the singular value directly as in the AMMIBS, it shrunk the cell means related to GEI at ratio of σe2σI2 where $\sigma_{I}^{2}$ is the GEI variance. The ratio of shrinkage obtained in our method is defined directly on the singular value on the ratio σe2σλk2 (S1 Appendix). Thus, in mixed models, the EBLUPs for GEI are function of a constant ratio of shrinkage, but in AMMIBS, different ratios are applied directly on singular value posterior showing the difference among the methods.

In the last decade, several methods for analyzing GE interactions in MET data have been proposed. Models that incorporate genotypes as random effects are being widely published and defended in the literature. The application of Bayesian methods, although not new [23,24], brings new prospects for MET data analysis. Using relatedness matrices replacing $\delta |\mu_{\delta },\sigma_{\delta }^{2}\sim N(0,I\sigma_{\delta }^{2})$ by $\delta |\mu_{\delta },\sigma_{\delta }^{2}\sim N(0,G)$ and heterogeneity of variance by replacing $I\sigma_{e}^{2}$ by *V* can be easily implemented and more informative priors can helps to generate more realistic estimates for the parameters of interest and should be a part of new research approaches.

This study illustrated that Bayesian shrinkage can be applied to AMMI models with relative success to select models and estimate parameters. However, the derivation of reference priors or maximum entropy for the parameters of the terms of the principal components may solve the problems of improper or even bimodal posteriors.

The results demonstrated that the traditional Bayesian AMMI model produces a small amount of shrinkage in the singular values and avoids problems determining credible intervals in the biplot. Bayesian shrinkage AMMI models have problems with the credible interval, but produce stronger shrinkage of the principal components, converging to more shrunken GE matrices than those obtained using mixed models. This characteristic allowed more parsimonious models to be chosen and more GEI pattern retained on the first two components. The resulting model chosen by posterior distribution of singular value was also similar to those produced by the cross-validation approach in traditional AMMI models. Our method enables the estimation of credible interval for AMMI biplot plus the choice of AMMI model based on direct posterior distribution retaining more GEI pattern in the first components and discarding noise without Gaussian assumption as requested in F-based tests or deal with parametric problems as observed in traditional AMMI shrinkage method.

## Supporting Information

S1 Appendix The AMMIBS model and the effect of shrinkage on the bilinear parameters. (DOCX)

S1 Table Cross validation based on leave-one-out approach. (DOCX)

S2 Table ANOVA for grain yield in 55 genotypes evaluated at 8 environments. (DOCX)

S1 Dataset Dataset. (CSV)

S1 Text R-code. (R)
