# Supplementary material for: A Bayesian Shrinkage Approach for AMMI Models
Source: PLoS One. 2015 Jul 9;10(7):e0131414. doi: 10.1371/journal.pone.0131414 (PMC4497624; doi:10.1371/journal.pone.0131414)
Supplement: S1 Appendix — (DOCX) [file pone.0131414.s001.docx]

# S1 Appendix

## The AMMIBS model and the effect of shrinkage on the bilinear parameters

The shrinkage estimator proposed by Cornelius and Crossa [8,11] to study GE interaction belongs to the class of shrinkage estimators proposed for *ridge regression* [35] and BLUPs [18,36], which are equivalent to Bayesian estimators with zero-mean normal priors. The shrinkage effect is a characteristic present in parameter estimators with random effects. However, studies performed by Cornelius and Crossa [11] showed that estimates obtained from the shrinkage method, for an AMMI model with fixed effects, are as good as or, in many cases, better than those obtained by BLUPs. Obviously, shrunken estimates are superior to least squares estimates, while avoiding the need for cross validation when selecting components for multiplicative models.

In this appendix, it is demonstrated that shrinkage of the parameter estimates that describe the GE interaction is a natural consequence when Bayesian approaches with the proper priors are used on singular values, i.e., this is a Bayesian interpretation of the method proposed by Cornelius and Crossa [11].

Based on the conditional posterior of the singular value, the posterior mean for $\lambda_{k}$ is as follows:

where is the number of replicates in the experiment and is the shrinkage factor for $\lambda_{k}$.

Rearranging the above expression yields the following:

Multiplying by

Adding and subtracting $\sigma_{e}^{2}$to the numerator yields:

Rearranging the above expression:

(15)

where is the mean effect of $\lambda_{k}$ in the Bayesian model suggested by Crossa.

Therefore, the shrinkage effect on $\lambda_{k}$ depends on the magnitude of $\sigma_{\lambda_{k}}^{2}.$As the expression approaches zero, the shrinkage of the mean effect of $\lambda_{k}$ becomes greater; similarly, as the value of $\sigma_{\lambda_{k}}^{2}$ increases, the shrinkage of the mean effect of $\lambda_{k}$ decreases.

Establishing a relation between the shrinkage factor with fixed effects and the shrinkage factor of the AMMIBS analysis, the factor established by Cornelius and Crossa [8] for the AMMI analysis is as follows:

$S_{k}=\frac{-u_{k}\sigma^{2}/n+E\left[\hat{\lambda}_{k}^{2}\right]}{E\left[\hat{\lambda}_{k}^{2}\right]}=\left(1-\frac{u_{k}\sigma^{2}}{nE\left[\hat{\lambda}_{k}^{2}\right]}\right)$ (16)

where $\frac{u_{k}\sigma^{2}}{nE\left[\hat{\lambda}_{k}^{2}\right]}$ is equivalent to an $F^{-1}$ test.

Similarly, the AMMIBS analysis in equation (15) can establish the same equivalence with an $F^{-1}$ test as follows:

(17)
