# Supplementary material for: A Bayesian Shrinkage Approach for AMMI Models
Source: PLoS One. 2015 Jul 9;10(7):e0131414. doi: 10.1371/journal.pone.0131414 (PMC4497624; doi:10.1371/journal.pone.0131414)
Supplement: S1 Table — (DOCX) [file pone.0131414.s002.docx]

## S1 Table. Cross validation based on leave-one-out approach

| **m** | **PRESS** | **W** | **PRECORR** |
| --- | --- | --- | --- |
| **1** | 0,5391 | 1,4256 | 0,9612 |
| **2** | 0,5207 | 0,2485 | 0,9627 |
| **3** | 0,5444 | -0,2731 | 0,9610 |
| **4** | 0,5130 | 0,3348 | 0,9635 |
| **5** | 0,4410 | 0,7558 | 0,9687 |
| **6** | 0,4248 | 0,1439 | 0,9698 |
| **7** | 0,4224 | 0,0163 | 0,9700 |
| **8** | 0,4224 | 0 | 0,9700 |
