# Supplementary material for: A Bayesian Shrinkage Approach for AMMI Models
Source: PLoS One. 2015 Jul 9;10(7):e0131414. doi: 10.1371/journal.pone.0131414 (PMC4497624; doi:10.1371/journal.pone.0131414)
Supplement: S2 Table — (DOCX) [file pone.0131414.s003.docx]

# S2 Table. ANOVA for grain yield in 55 genotypes evaluated at 8 environments.

|  | **Df** | **Sum Sq** | **Mean Sq** | **F value** | **Pr(>F)** |  |
| --- | --- | --- | --- | --- | --- | --- |
| Environments | 8 | 8994.2409 | 1124.2801 | 964.1083 | 2.00e-16 | *** |
| Genotypes | 54 | 593.484 | 10.9904 | 9.4247 | 2.00e-16 | *** |
| Blocks(Env.) | 18 | 57.4934 | 3.1941 | 2.7390 | 0.000127 | *** |
| Env. x Genotype | 432 | 938.1086 | 2.1715 | 1.8622 | 1.82e-15 | *** |
| Residuals | 972 | 1133.4829 | 1.1661 | - | - |  |
| Total | 1466 | 11715.8098 | - | - | - |  |
